# First description of deep benthic habitats and communities of oceanic islands and seamounts of the Nazca Desventuradas Marine Park, Chile

**DOI:** 10.1038/s41598-021-85516-8

**Published:** 2021-03-18

**Authors:** Jan M. Tapia-Guerra, Ariadna Mecho, Erin E. Easton, María de los Ángeles Gallardo, Matthias Gorny, Javier Sellanes

**Affiliations:** 1 Programa de Magister en Ciencias del Mar Mención Recursos Costeros, Facultad de Ciencias del Mar, Universidad Católica del Norte, Larrondo 1281, Coquimbo, Chile; 2 Sala de Colecciones Biológicas, Facultad de Ciencias del Mar, Universidad Católica del Norte, Larrondo 1281, Coquimbo, Chile; 3 Millennium Nucleus for Ecology and Sustainable Management of Oceanic Islands (ESMOI), Departamento de Biología Marina, Facultad de Ciencias del Mar, Universidad Católica del Norte, Larrondo 1281, Coquimbo, Chile; 4 UMR8212 Laboratoire des Sciences du Climat et de l’Environnement (LSCE), Paris, France; 5 School of Earth, Environmental, and Marine Sciences, University of Texas Rio Grande Valley, 33363 Marine Lab Dr., South Padre Island, TX 78597 USA; 6 Oceana Inc. Chile, Santiago, Chile

**Keywords:** Ichthyology, Biodiversity, Biogeography, Conservation biology, Marine biology, Ecology, Zoology, Ocean sciences

## Abstract

Seamounts and oceanic islands of the Chilean Exclusive Economic Zone at the intersection of the Nazca and Salas y Gómez ridges lie within one of the least explored areas in the world. The sparse information available, mainly for seamounts outside Chilean jurisdiction and shallow-water fauna of the Desventuradas Islands, suggests that the area is a hotspot of endemism. This apparent uniqueness of the fauna motivated the creation of the large Nazca-Desventuradas Marine Park (NDMP, ~ 300,000 km^2^) around the small islands San Felix and San Ambrosio in 2015. We report for the first time a detailed description of benthic microhabitats (i.e., centimeter to meter scale), macrohabitats (i.e., meter to kilometer-scale) and associated megafauna within the NDMP. Descriptions were based on analysis of fauna collected by trawling and ROV video observations from ~ 50 to 370 m depth. Rocky, coarse sand and silty sediment bottom habitats were observed at island slopes. In contrast, rocky and coarse sandy bottom habitats with a predominance of rhodoliths, thanatocoenosis, and other biogenic components were observed at seamounts. Mobile fauna and predators dominated the oceanic islands and nearby seamounts, whereas seamounts farther from the islands were dominated by sessile and hemisessile fauna that were mainly suspension and deposit feeders. Based on the register of 118 taxonomic units, our results provide an expanded and updated baseline for the benthic biodiversity of NDMP habitats, which seemed pristine, without evidence of trawling or anthropogenic debris.

## Introduction

Seamounts are topographic elevations that rise more than 1000 m above the seafloor^1^, and in some cases, they reach the euphotic zone or the surface (i.e., oceanic islands)^2^. These ecosystems are considered vulnerable marine habitats because they are physically fragile or inherently rare^3, 4^. Studied seamounts and oceanic islands are generally considered to play important roles by being highly productive locally, acting as regional centers of speciation or stepping-stones for dispersion. They comprise a diversity of substrates and thus habitats that support and provide refuge and feeding areas, not only for the benthic fauna but also for the associated pelagic and overlying surface water animals (e.g., birds, and marine mammals)^5–7^. Although seamounts are generally considered to play these important roles, each seamount experiences unique environmental conditions such as current characteristics and water-column environmental conditions (i.e., temperature, salinity and pressure) that differ with depth and current dynamics^6^. They also have unique characteristics such as height and morphology that result in unique physical dynamics that can result in increased nutrient availability relative to the surrounding area (reviewed in^6^). Such variability in environmental conditions results in variable ecological characteristics. Despite the above-mentioned importance of seamounts, only 4% of the ~ 40,000 reported have been sampled for scientific purposes^8–10^. The available faunistic information for seamounts is limited (especially for depths > 300 m) and, for unstudied seamounts, usually comes from commercial fishing activities^11^.

The Pacific Ocean basin is characterized by the existence of several relatively long quasi-linear chains of oceanic islands and seamounts^12^. The oceanic islands and seamounts of the southeast Pacific lie within one of the most unexplored areas in the world. Most of the ~ 940 southeast Pacific seamounts are located along the Salas y Gómez (SGR) and Nazca (NR) ridges^13, 14^, and only 24 (< 3%) of the SGR and NR seamounts have been studied. Most of these studies were conducted by expeditions of the former USSR between 1973 and 1987 in international waters from ~ 80°W to 101°W at 162 to 1900 m depth^15^. They reported unprecedented rates of endemism for benthic communities (e.g., ~ 41% for fishes and ~ 46% for invertebrates) that were similar to those rates observed for seamounts of the remote Norfolk Ridge, near New Caledonia^16^. Despite studies being conducted on SGR and NR seamounts^15, 17–19^, only one of the many seamounts within the Chilean EEZ adjacent to the Desventuradas Islands (San Felix and San Ambrosio islands) has been investigated. In addition to this seamount, known as Stockman Guyot^11, 15^, the Desventuradas Islands were studied on two expeditions: Marine Research Cruises in Remote Areas (CIMAR 6) conducted by the National Oceanographic Committee of Chile (CONA) in 2001 and the "Pristine Seas Expedition" carried out by the National Geographic Society and Oceana in 2013. These studies, which were conducted at 40 to 2300 m^18, 20^, revealed that the fauna of these islands are unique and irreplaceable, granting these areas a high conservation value^20, 21^. These considerations prompted the creation, in 2015, of the large Nazca Desventuradas Marine Park (NDMP), protecting an area of ~ 300,000 km^2^ including the Desventuradas Islands and seamounts located northwest of them^22^. This marine park provides enormous advances in safeguarding the unique biodiversity of actual or past threats such as pelagic fishing of tuna and swordfish, as well as bottom trawling^7^.

Of the available studies in the region, none has explored the importance of substrate type on species composition^23^, nor the role of the habitat at different scales from centimeters to meters. Because distinct environmental conditions, such as internal tides and heterogeneity of substrates and oceanographic conditions (i.e., temperature, salinity and oxygen gradients), can differ considerably among and within seamounts^24^, these and other variables can play a critical role in the distribution and behavior of megabenthos, operationally defined here as organisms with a body size > 1 cm. Differences in these environmental conditions can generate a mosaic of micro- and macrohabitats, with breaks in species distributions that can enhance biodiversity and drive changes in community composition within or among seamounts^23, 24^.

The heterogeneity and topographic complexity of seamounts is affected by the presence of organisms that can change spatial conditions and, directly or indirectly, affect the availability of habitat and resources for other species^25^. These organisms are called habitat-forming species and are characterized by being small-scale engineers of autogenous ecosystems. These organisms, and the biogenic structures they produce, contribute to increasing spatial complexity in soft-sediment benthic systems^25^, promoting spatial heterogeneity on seamounts^26^, but they are often easily overlooked because seamounts are sampled at much more general spatial scales^26^. In recent years, the importance of sampling at smaller scales has been accentuated with the understanding that the characteristics of the bottom and the presence of habitat-forming species increases the heterogeneity of the habitat and fulfill the function of a structural habitat^27^.

Several studies of continental shelf, slope and submarine canyon habitats have specified relationships of megafauna with certain microtopographic (microhabitat) characteristics (e.g., depressions, burrows and sessile fauna)^28–30^. Because many of these studies lacked a standard habitat classification scheme, these observations cannot be compared efficiently among ecosystems and studies. To explore the importance of seabed habitat, Greene et al.^31^ proposed a standardized classification, which divides the habitat according to depth, size and biogenic and abiotic variables, for in situ observations of the seabed. Within the size category are the subcategories of macrohabitat and microhabitat. Macrohabitats range in size from one to ten meters and include seafloor features such as boulders, crevices, cracks, caves, scarps, sinkholes and bedrock corals (solitary and reef-building). Meanwhile, microhabitats operate at centimeters and smaller scales and include seafloor features such as sand, silt, gravel, pebbles, small cracks, crevices and fractures^31, 32^.

To explore relationships among micro- and macrohabitats and the fauna living on the summit of the seamounts and upper slope of oceanic islands of the NDMP, we describe the benthic habitats and associated fauna of the upper slope (50–370 m) of Desventuradas Islands and the summits and upper slopes (150–305 m) of seven nearby seamounts. We conducted remotely operated vehicle (ROV) surveys complemented with benthic fauna collections using an Agassiz trawl, aiming to achieve the following objectives: (1) to describe the benthic habitats of the oceanic islands and seamounts of the NDMP at different spatial scales (i.e. at centimeter to meter scale), (2) to describe the composition and the diversity of the benthic megafauna on these oceanic islands and seamounts, and (3) to evaluate the relationships between macrohabitat and megafauna species diversity and composition as well as feeding mode and movement type of the benthic communities. To our knowledge, this study is the first to describe benthic habitats and associated fauna at seamounts and oceanic islands of the NDMP.

## Materials and methods

### Study area

**Figure 1:**
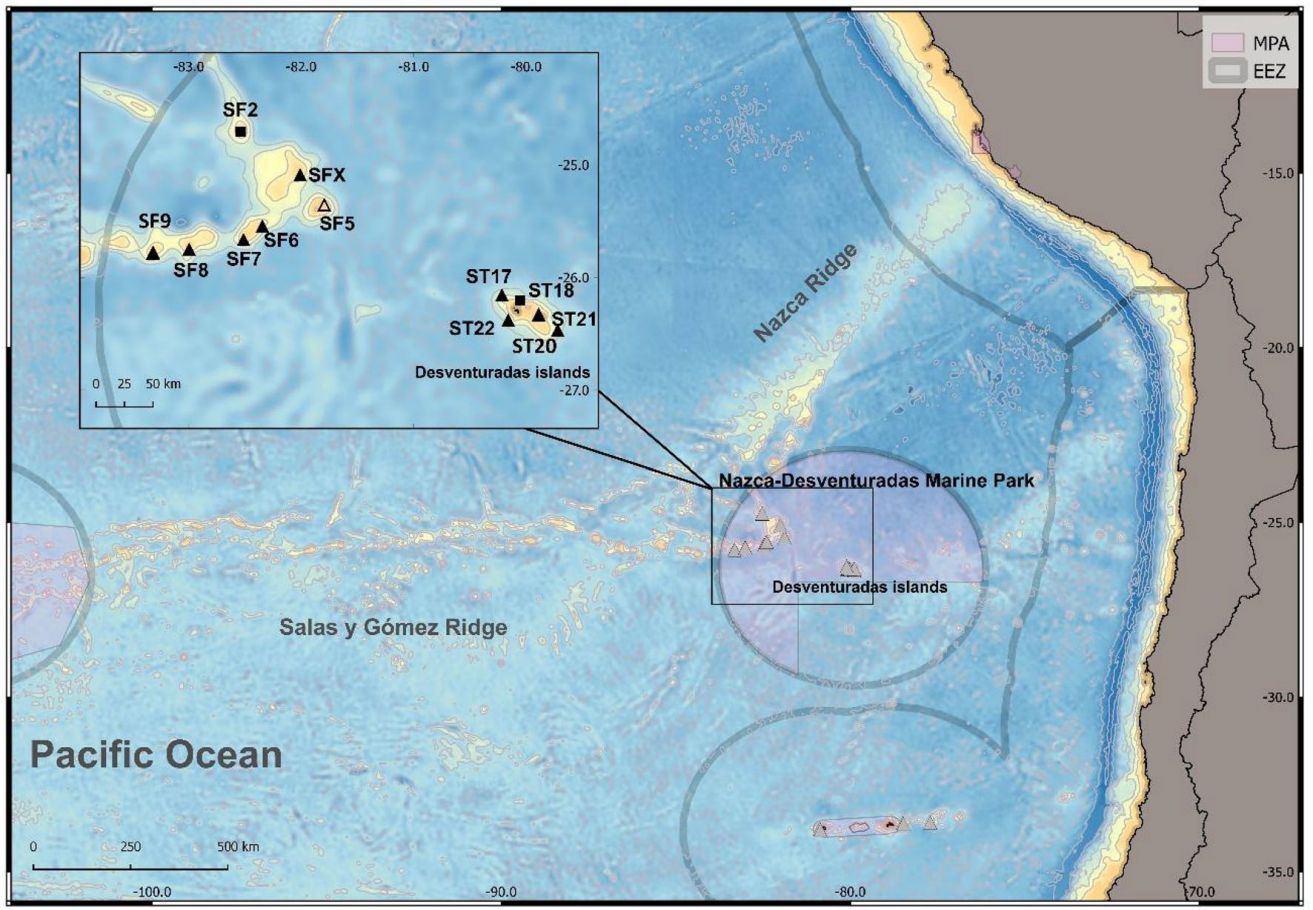
Location of the benthic stations sampled during CIMAR 22 cruise (13 October to 14 November 2016). Locations with Agassiz trawls and ROV deployments (filled upward triangle), only ROV deployments (filled square), and only Agassiz trawls (white upward triangle). Map generated using QGIS (version 3.10)^72^.

The NR and SGR together form a sequential chain of seamounts of volcanic origin with an extension of ~ 2900 km^33^ that is ~ 100 km wide along the SGR^34^ and ~ 300 km wide along the NR^35^. The Desventuradas Islands, constituted by San Felix and San Ambrosio islands (~ 26°S, 80°W), are located ~ 970 km off the coast of the Atacama Region, northern Chile. Seamounts of the Desventuradas Islands region are located northwest of these islands at the intersection of SGR and NR (Fig. 1)^36^.

### Data collection

From 22 October to 1 November 2016, benthic surveys were performed at five stations on the upper slope of the Desventuradas Islands and the summits of seven nearby seamounts of the NR (Fig. 1). A Commander MK2 (Mariscope Meerestechnik, Kiel, Germany) ROV equipped with an HD Camcorder (Panasonic SD 909, resolution 1920 × 1080, 30 fps), a GoPro Hero camera (resolution 2704 × 1520, 60 fps) and laser pointers (10 cm apart) was used to survey benthic habitats and communities. A total of 11 ROV dives were conducted at ~ 40 to 370 m depth (Table S1); five on the slope of Desventuradas Islands (5 h of total bottom time) and six on the seamounts (6 h of total bottom time). The ROV observations were descriptive and did not aim to obtain quantitative data due to the potential overlapping of the paths of the surveys.

Benthic trawling was performed at stations where either multibeam or ROV observations indicated suitable soft bottoms. Trawls were conducted for ~ 4 to 15 min (bottom contact) with a constant speed of ~ 3 knots at each station (Table S1). The gear used was a modified Agassiz trawl, with a mouth of 1.5 m × 0.5 m (width × height) fitted with a net of 12 mm mesh at the cod end. A total of 10 trawls were conducted; four on the slope of the Desventuradas Islands and six on the seamount summits, with sampling depths ranging from 133 to 340 m (Table S1). Both trawl and ROV deployments were possible at all stations, except at ST18 and SF2 (only ROV data because the bottom was too rocky to safely conduct trawls) and at SF5 (only trawl data because strong currents prohibited ROV deployment) (Fig. 1).

Environmental data (i.e., temperature, salinity and oxygen) used in our analysis were obtained from the global three-dimensional CSIRO Atlas of Regional Seas (CARS) climatology (2009 version) as presented by Mecho et al.^37^.

### Habitat description based on ROV videos

The oceanic islands (OI) and seamounts (SM) were considered as two different environments (corroborated by statistical analyses presented below) and were referred to as subsystems, based on differences in physiography and depth. The characteristics of the seafloor habitats for each subsystem were described based on ROV data. Videos for each dive were analyzed in a time-lapse mode at half normal speed in VLC media Player 3.0.11^38^. Habitats were classified at different spatial scales: (1) macrohabitats (i.e., meter-scale) and (2) microhabitats (i.e., centimeter-scale). The habitat types were described by geomorphology, including by slope (i.e., flat = 0°–5°, sloping = 5°–30°, steeply sloping = 30°–60°, vertical = 60°–90°), sediment type (i.e., rock, silty sediments, mixed substrate, coarse sand), texture (i.e., low rugosity, moderate rugosity, high rugosity) and "modifier" elements (i.e., biological communities, sedimentation and bio-perturbation) following Greene et al.^31, 32^. The sections of the videos in which the ROV was steadily moving between 30 cm and 1 m above the ground along the bottom were used for the description of the macrohabitats (field of view per frame ~ 3 m^2^), and the sections in which the ROV remained sitting on the bottom (field of view per frame ~ 0.05 m^2^) were used for the description of the microhabitats. The field of view per frame was estimated using the laser pointers of the ROV (positioned 10 cm apart).

### Benthic and demersal fauna description

Specimens collected with the Agassiz trawl were preliminarily sorted, counted, and preserved onboard in 95% ethanol. Definitive counts and identification to the lowest possible taxonomic unit (hereafter operational taxonomic units, or OTUs) were performed at Sala de Colecciones Biológicas Universidad Católica de Norte (SCBUCN), where specimens were then assigned an ID number and cataloged. The observed fauna was classified according to relevant literature and previous reports for the area (e.g.,^15, 39–45^). Taxonomic assignments were further validated with resources such as the World Register of Marine Species (Worms, 2020. http://www.marinespecies.org) and the Ocean Biodiversity Information System (OBIS, 2020, Intergovernmental Oceanographic Commission of UNESCO. www.iobis.org).

Semi-quantitative data of abundance for each OTU were calculated based on the swept area for each trawl. The swept area (Ab_i_) was established as the product of the trawling speed (V_i_), effective trawl time (t_i_) and width of the mouth of the trawl (Ah_i_) and abundance was standardized to 10 m^2^ following Barriga et al.^46^:

$${Ab}_{i}={t}_{i}{V}_{i}{Ah}_{i}$$

The ROV recordings followed an exploratory methodology which restricted the data to the presence/absence of OTUs at each station. Videos for each transect were viewed at half their normal speed in VLC. The VLC tool "Interactive Zoom" was used to magnify images to observe the diagnostic characteristics of the taxa. Observations that were blurry or too distant were omitted because they did not provide sufficient details for identification.

All the sampling was performed under permission Res. Ext N°3685/2016 from SUBPESCA (National Fishing Authority of Chile) to Universidad Católica del Norte.

### Data analysis

All analyses were performed using the R software, version 4.0.3^47^ using the “vegan v2.5-6” package^48^. Because both trawl and ROV data were not available for all stations (Table S1), the analysis approach considered either only trawl data or combined trawl and ROV data as follows. Only trawl data was used for the estimation of Shannon diversity (*H*′) and Pielou’s evenness (*J*) since abundance data is required. OTU richness (or total *S*) of each station was estimated based on the combined trawl and ROV data (except for ST18, SF2 and SF5 stations). In all cases, only "living" organisms were considered (i.e., empty shells or skeletons excluded) for the analyses. Species accumulation curves were constructed for each subsystem (OI and SM) and data gathering approaches (only trawl and combined trawl and ROV data) to estimate the rarified number of species and to assess sampling effort^48^. The OTU richness and diversity indexes of the two subsystems (OI and SM) were compared using an unpaired two-sample Wilcoxon test.

To evaluate if there were differences in OTU composition among subsystems and stations a permutational analysis of variance (PERMANOVA), based on combined trawl and ROV data (ST18, SF2 and SF5 stations were excluded), was conducted. The routine “adonis2” on the Bray–Curtis index of similarity of the raw OTU presence/absence data (10,000 runs) with subsystems (two levels: OI and SM) and maximum depth as factors was used. Patterns in the structure of benthic communities among subsystems and stations were visualized using a cluster analysis UPGMA hierarchical clustering (using the functions “desvest” and “hclust”) and non-metric multidimensional scaling (nMDS; using the function “metaMD”) analyses based on Bray–Curtis dissimilarity elaborated from OTU presence/absence data. To analyze differences in functional diversity among sampling stations, OTUs were assigned to feeding modes (i.e., suspension, depositor, grazer, opportunistic or predator) and movement type (i.e., sessile, burrower/tube dweller, crawler or swimmer) based on functional traits and associated categories as proposed by Jones & Frid^49^. A canonical correspondence analysis (CCA) was applied using the R function “cca” to relate the set of environmental parameters to: (1) OTUs presence/absence data, (2) feeding mode composition, and (3) movement type. Environmental variables included water hydrographic variables (temperature and oxygen), type of bottom (rock, mud and sand), depth, and geographic position (longitude and latitude). Salinity was not considered within the environmental variables in the nMDS and CCA because it co-varies with the oxygen. nMDS and CCA analysis were graphed using the library ggplot2 v3.3.2 in R^50^.

## Results

### Description of macrohabitats

**Figure 2:**
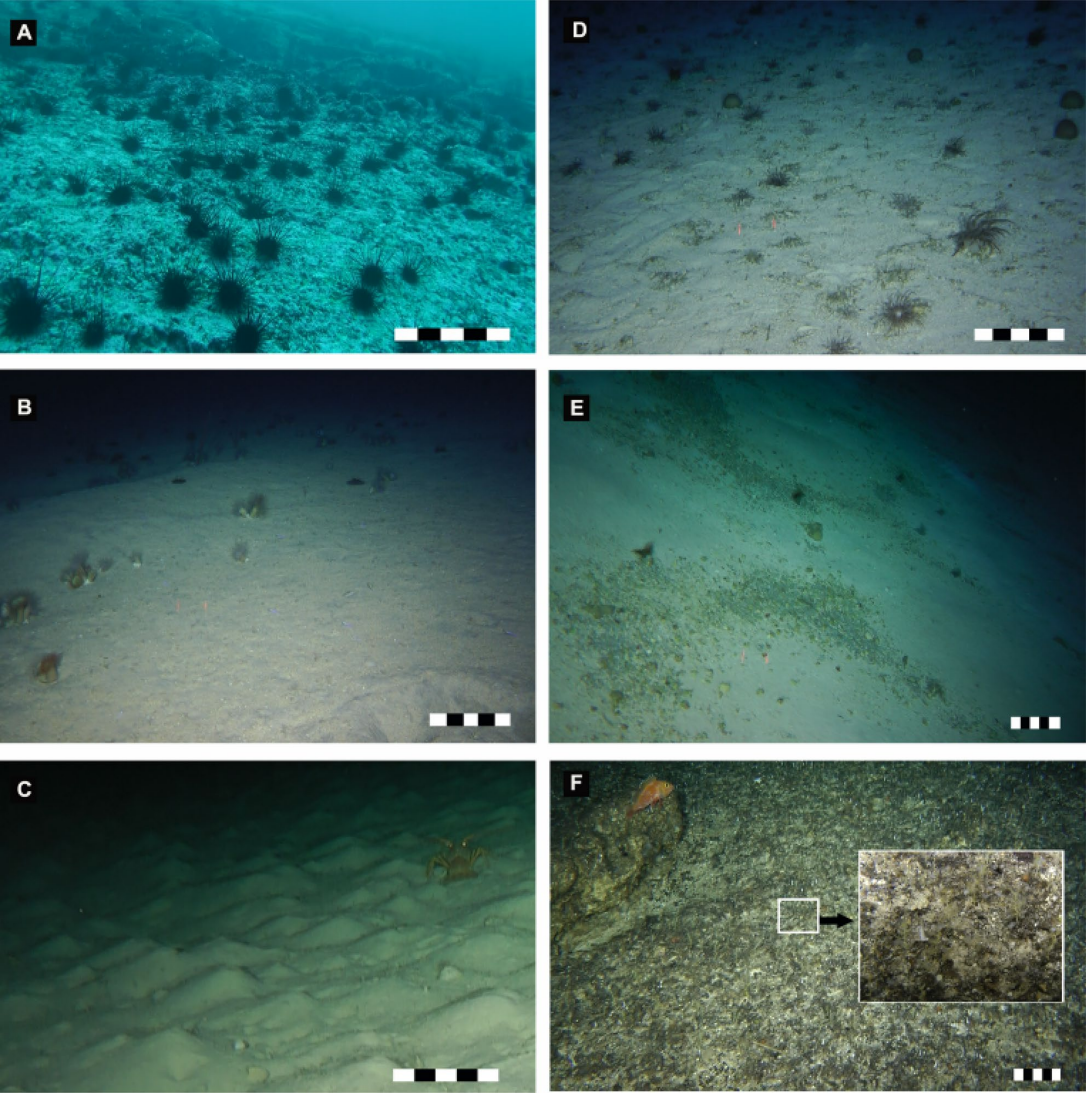
Benthic macrohabitats on the upper slope of Desventuradas islands (**A**–**C**) and summits of seamounts (**D**–**F**) at the Nazca-Desventuradas Marine Park. (**A**) Irregular rock bottom with fractures and faults, (**B**) regular continuous homogeneous bottom with coarse sand dominated by anemones (*Hormathia* sp), (**C**) silty sediments with the presence of bioturbation ripples, (**D**,**E**) regular continuous homogeneous bottom with little relief, coarse sand and rhodoliths, dominated by sponges (unidentified demosponges) and anemones (*Hormathia* sp. and cerianthids), (**F**) irregular rock bottom with structures fractured, faulted and folded, characterized by sea pens (*Scleroptilum* sp.), hydrozoan corals (*Stylaster* sp.) and some fishes (*Helicolenus lengerichi*). Scale bar: 25 cm. Image credits: ESMOI/OCEANA.

**Figure 3:**
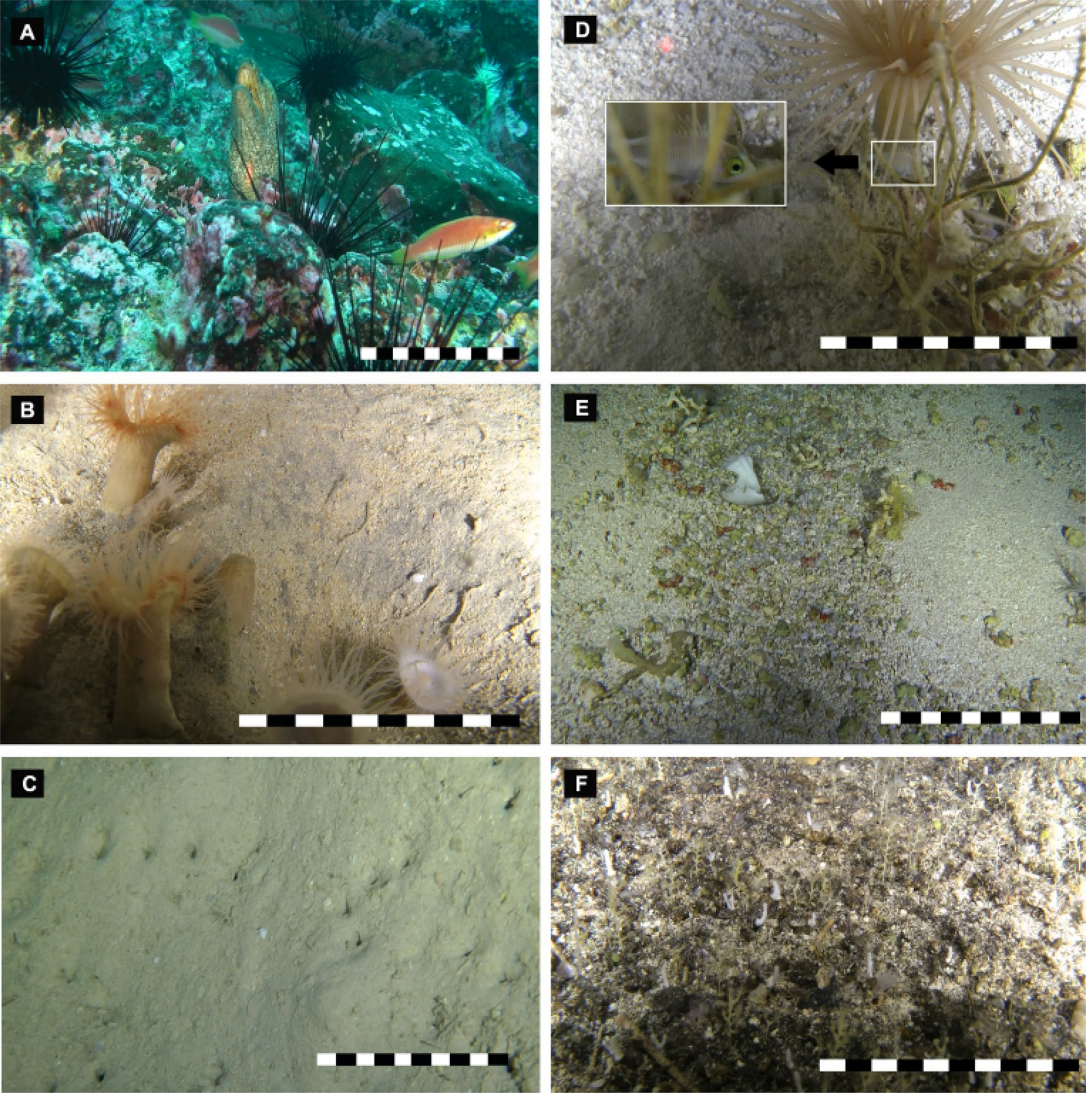
Benthic microhabitats on the upper slope of Desventuradas islands (**A**–**C**) and summits of seamounts (**D**–**F**) at the Nazca-Desventuradas Marine Park. (**A**) Rocky bottom dominated by incrusting red algae, sea urchin *Centrostephanus sylviae*, the cracks and faults of the rocky bottom, are used as habitats for moray eels (*Gymnothorax porphyreus*) and small fishes (*Pseudolabrus* cf. *gayi*), (**B**) coarse sand flat bottom dominated by anemones (*Hormathia* sp.), (**C**) soft-sediment with burrows and excavations, (**D**) microhabitats of cerianthid anemones and hydrozoan colonies, (**E**) unattached nodules of crustose coralline red algae (Rhodoliths), demosponges, and coquina of pteropod shells, (**F**) irregular rock bottom with structures fractured, faulted and folded, characterized by sea pens (*Scleroptilum* sp.) and hydrozoan corals (*Stylaster s*p.). Scale bar: 10 cm. Image credits: ESMOI/OCEANA.

Oceanic island macrohabitats (~ 43 to 370 m depth) were classified to one of three types (Table 1): (1) cobbles and bedrocks, predominated by sea urchins (i.e., *Centrostephanus sylviae*, Fig. 2A), (2) coarse sand, with anemones (mainly *Hormathia* sp.) (Fig. 2B), and (3) silty sediments with the presence of bioturbation (e.g., burrows) ripples with a maximum height of ~ 10 cm (Fig. 2C).

**Table 1:** Description of benthic habitats of the oceanic islands (Desventuradas).

Stations	Subsystem	Class (macrohabitat)	Subclass (microhabitat)	Environmental variables	Modifiers
ST17	OI	Flat bottom	Slope: flat (0–5°) Texture: smooth surface Sediment types: mixed sediment, coarse sand	Salinity: 34.4 Temperature (C°): 13.9 Oxygen (mL/L): 5	Bottom morphology: regular-continuous homogeneous bottom with little relief with organic debris (coquina) Biological processes: conspicuous microhabitats/communities of anemone, sea pens and kelps patch
ST18	OI	Sloping bottom, with vertical sections	Slope: steeply sloping (30°–60°) Texture: moderate rugosity to high rugosity surface Sediment types: bedrock and boulde	Salinity: 34.7 Temperature (C°): 17.7 Oxygen (mL/L): 5.5	Bottom morphology: irregular bottom with structures fractured, faulted and folder Bottom texture: occupied by clast or rock Biological processes: conspicuous microhabitats/communities of coralline incrusting algae, sponge and anemone
ST20	OI	Type I: steep slope bottom Hard substrates and steep slope of compact cemented rock Type II: flat bottom	Type I Slope: sloping (5°–30°) Texture: moderate rugosity Sediment types: bedrock and boulder Type II Slope: flat (0°–5°) Texture: smooth surface Sediment types: mixed sediment, coarse sand	Salinity: 34.3 Temperature (C°): 12.9 Oxygen (mL/L): 4.7	Type I Bottom morphology: irregular bottom with structures fractured, faulted and folder Bottom texture: occupied by clast or rock Biological processes: conspicuous microhabitats/communities of coralline incrusting algae, sponge and anemone Type II Bottom morphology: regular-continuous homogeneous bottom with little relief Biological processes: conspicuous microhabitats/communities of anemones and sea pens
ST21	OI	Flat bottom	Slope: flat (0°–5°) Texture: smooth surface Sediment types: mixed sediment, coarse sand	Salinity:34.3 Temperature (C°): 12.9 Oxygen (mL/L): 4.7	Bottom morphology: regular-continuous homogeneous bottom with little relief Biological processes: conspicuous microhabitats/communities of anemones and sea pens
ST22	OI	Sloping bottom with bedform-sediment waves	Slope: sloping (5°–30°) Texture: very low rugosity Sediment types: silty sediments	Salinity: 34.5 Temperature (C°): 8.4 Oxygen (mL/L): 1.3	Bottom morphology: irregular bottom (Undulated surface -ripples) with sediment waves (10 cm to amplitude) Bioturbation: burrows and excavations
SF2	SM (top)	Steep slope bottom hard substrates and steep slope of compact cemented rock	Slope: steeply sloping (30°–60°) Texture: moderate rugosity to high rugosity surface, rock bottom	Salinity: 34.5 Temperature (C°): 10.4 Oxygen (mL/L): 1.6	Bottom morphology: rock bottom with smalls accumulation of coarse sand Biological processes: conspicuous microhabitats/communities of sponges, sea pens and white corals
SF6	SM (top)	Flat bottom	Slope: flat (0–5°) Texture: smooth surface Sediment types: mixed sediment, coarse sand	Salinity: 34.3 Temperature (C°): 13.5 Oxygen (mL/L): 4.4	Bottom morphology: regular- continuous homogeneous bottom with little relief Biological processes: conspicuous microhabitats/communities of anemones, hydrozoan colonies, biocenosis (sea urchin skeletons)
SF7	SM (top)	Flat bottom	Slope: Flat (0–5°) Texture: smooth surface Sediment types: mixed sediment, coarse sand	Salinity: 34.4 Temperature(C°): 13.5 Oxygen (mL/L): 4.4	Bottom morphology: regular- continuous homogeneous bottom with little relief Biological processes: conspicuous microhabitats/communities of anemones, rhodoliths, sponges, thanatocoenosis (coquina- Pteropod shells)
SF8	SM (top)	Flat bottom	Slope: Flat (0–5°) Texture: smooth surface Sediment types: mixed sediment, coarse sand	Salinity: 34.4 Temperature (C°): 10.3 Oxygen (mL/L): 2.1	Bottom morphology: regular- continuous homogeneous bottom with little relief Biological processes: thanatocoenosis (coquina- bivalve shells); Bioturbation (burrows and excavations)
SF9	SM (top)	Flat bottom	Slope: Flat (0–5°) Texture: smooth surface Sediment types: mixed sediment, coarse sand	Salinity: 34.2 Temperature (C°): 10.3 Oxygen (mL/L): 4.3	Bottom morphology: regular- continuous homogeneous bottom with little relief Biological processes: conspicuous microhabitats/communities of anemones, rhodoliths, sponges, thanatocoenosis (coquina- Pteropod shells)
SFX	SM (top)	Flat bottom	Slope: Flat (0–5°) Texture: smooth surface Sediment types: mixed sediment, coarse sand	Salinity: 34.4 Temperature (C°): 11.5 Oxygen (mL/L): 2.6	Bottom morphology: regular- continuous homogeneous bottom with little relief Biological processes: conspicuous microhabitats/communities of anemones, amphinomid polychaetas and detrital patch

Classification follows Greene et al.^1^ and Greene et al.^32^. Subsystem OI = oceanic islands (upper slope) and SM = seamounts (top).

Seamount macrohabitats (~ 150 to 305 m depth), except at SF2, consisted of coarse sand and rhodolith beds (Fig. 2D,E, Table 1) with large patches of anemones and sea pens. Seamount SF2 substantially differed from the others by the predominance of hard substrates and a steep slope of compact cemented rock, with small patches of coarse sand and large rock formations. The sessile fauna of seamount SF2 consisted of a multitude of hydrozoans, sea pens, and small corals, among other sessile fauna associated with rocky substrates. Also, the presence of longitudinal trenches in the cemented rock gave shelter to numerous fishes (e.g., *Lotella* cf. *fernandeziana*, *Helicolenus lengerichi* and *Scorpaena thomsoni*) and large crustaceans (e.g., the Chilean jagged lobster *Projasus bahamondei* and the Juan Fernández carrier crab *Paromola rathbuni*) (Fig. 2F).

### Description of microhabitats

Oceanic island microhabitats were classified into one of three types (Table 1): (1) joints, cracks, crevices, and overhangs (differentially eroded) covered by incrusting algae, sponges and stony corals (Fig. 3A) at 43–50 m; (2) coarse sand with sea pens and anemone colonies, which provide harbor for fishes and invertebrates at 128–150 m (the most common habitat type observed on oceanic islands) (Fig. 3B), and (3) silty sediment bottom, absence of epibenthic cnidarians (e.g., anemones or sea pens). Besides, type 3 microhabitat was observed to have small holes on the surface of the sediment at 220–370 m, possibly caused by infaunal excavators. (Fig. 3C).

Cnidarians and sponges are the most conspicuous biological components of seamount microhabitats (~ 150 to 305 m depth), which included fields of sea pens (*Protoptilum* sp. and *Scleroptilum* sp.) and colonies of anemones (*Hormathia* sp. and Cerianthid), except at SF2. Besides, we also reported biogenic structures on coarse sand beds such as coquina of pteropod (Fig. 3D) and rhodoliths (Fig. 3E). Although the SF2 seamount is characterized by a hard and fractured substrate (faults and folds) covered with small corals (*Stylaster* cf. *marenzelleri*) and green sponges, small patches of coarse sand were observed between the rocks with sea pens of the genus *Scleroptilum* (Fig. 3F).

### Benthic community

In total, 2414 individuals (only living specimens) were collected by trawling and assigned to 95 OTUs (OI: 44 OTUs and SM: 75 OTUs, 24 OTUs shared between subsystems), belonging to nine phyla (Table S2). In terms of total abundance, Cnidaria was the predominant major faunal group, comprising 29% of the total capture, followed by Porifera (26%), Arthropoda (13%), Echinodermata (13%), Mollusca (7%) Annelida (7%), Sipuncula (5%), Chordata (1%) and Bryozoa (0.2%). ROV observations resulted in a total of 61 OTUs (OI: 46, SM: 38, shared between subsystems: 23), of which 23 OTUs were observed only with this approach, 17 of these 23 OTUs were fish.

**Figure 4:**
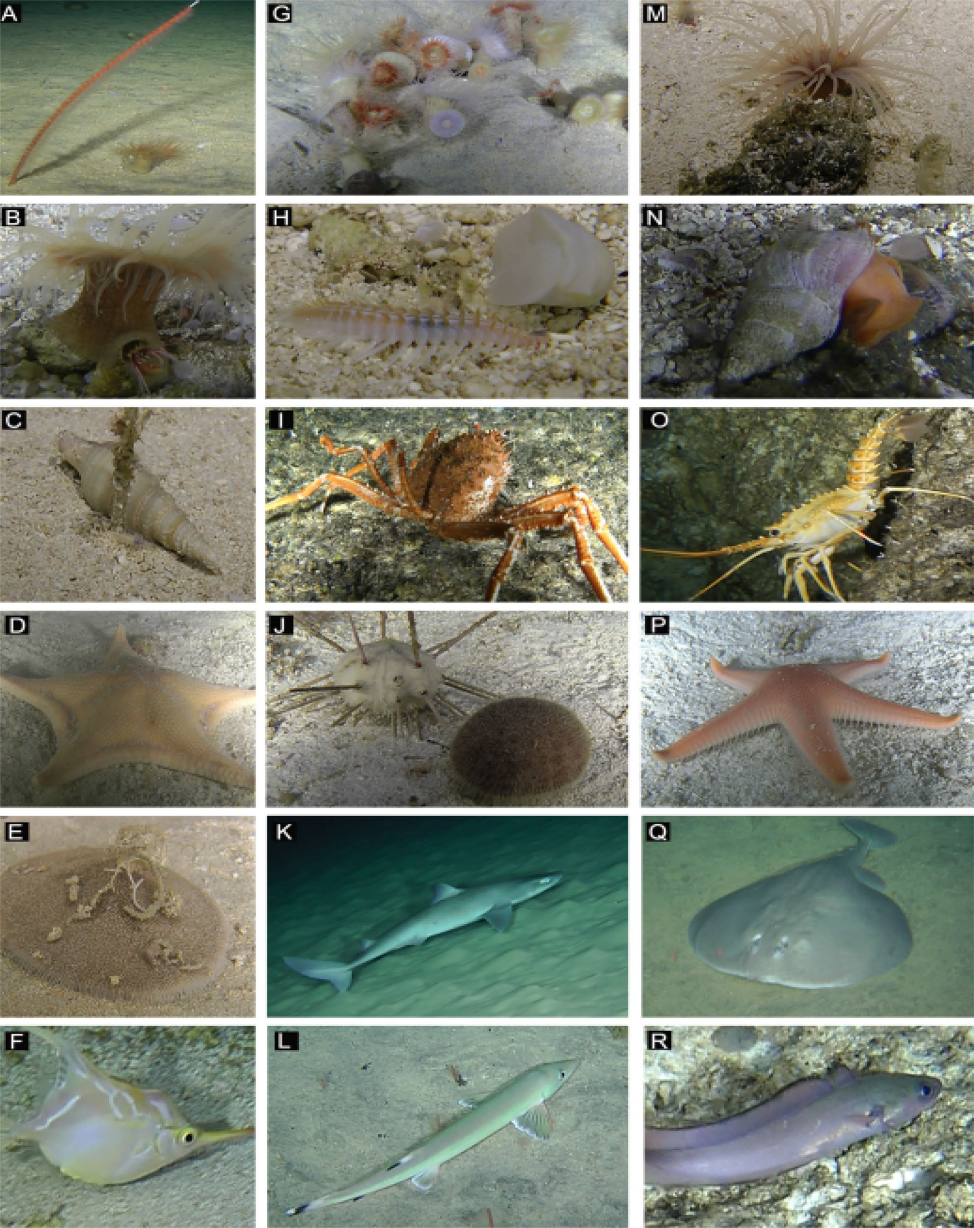
Representative OTUs observed at the upper slope of Desventuradas islands and seamounts within the Nazca-Desventuradas Marine Park. (**A**) Sea pen *Protoptilum* sp. and anemone *Hormathia* sp., (**B**) anemone (*Hormathia* sp.) and hermit crab *Paragiopagurus boletifer*, (**C**) *Cryptogemma praesignis*, (**D**) *Anthenoides* sp., (**E**) *Clypeaster isolatus*, (**F**) *Notopogon fernandezianus*, (**G**) anemone colony *Hormathia* sp., (**H**) *Chloeia* sp., (**I**) *Paromola rathbuni*, (**J**) *Stereocidaris nascaensis* (left) and *Scrippsechinus fisheri* (right), (**K**) *Squalus mitsukurii*, (**L**) *Gonorhynchus greyi*, (**M**) tube anemone Ceriantharidae, (**N**) *Chryseofusus kazdailisi*, (**O**) *Projasus bahamondei*, (**P**) *Pseudarchaster* sp., (**Q**) *Tetronarce* sp., and (**R**) *Lotella fernandeziana*. Laser pointers, when visible, are 10 cm apart. Image credits: ESMOI/OCEANA.

Combining collected specimens and ROV observations allowed allocation of a total of 118 OTUs (OI: 65, SM: 84, and shared between subsystems: 31). An additional 13 OTUs were identified just by shells or skeleton remains (e.g., pteropod or other mollusk shells), but were not included in the analyses. Invertebrates represented 75% of the OTUs, whereas vertebrates represented 25% (Fig. 4). Chordata (25%), Arthropoda (23%), Mollusca (13%) and Echinodermata (13%) were the most OTU-rich taxa in the total *S* data. Invertebrates were assigned to eight phyla: Arthropoda (23%), Mollusca (13%), Echinodermata (13%), Cnidaria (12%), Annelida (10%), Porifera (3%), Sipuncula (1%), Bryozoa (1%). Vertebrates (Chordata) were assigned to two classes of fishes (Table S2): Elasmobranchii (2 OTUs) and Actinopterygii (28 OTUs). In terms of overall OTU richness, fishes predominated at Desventuradas Islands (43%) and arthropods at seamounts (30%).

Combined OTU richness (total *S* data) for oceanic islands was highest at station ST20 (*S* = 35), representing almost half of the total OTUs reported at the oceanic islands, followed by station ST21 (*S* = 15). The lowest OTU richness at oceanic islands was observed for station ST18 (*S* = 10) (Table 2). OTU richness at seamounts was highest at station SF9 (*S* = 50), which represents ~ 40% of the total OTUs reported for seamounts, followed by station SF7 (*S* = 35). The lowest OTU richness was observed at seamount stations SF8 (*S* = 8) and SFX (*S* = 13) (Table 2). However, no differences in total *S* (Wilcoxon test, W = 6.5, *p* = 0.284) or diversity (H '; Wilcoxon test, W = 8, *P* = 0.456) were observed between subsystems. Stations at oceanic islands, in general, were less diverse (*H*′ = 1.7, mean) in comparison to the seamounts (*H*′ = 1.9). The diversity for oceanic islands was highest at station ST20 (H′ = 2.5) followed by station ST22 (*H*′ = 1.6). The lowest diversity at oceanic island stations was observed at ST21 (*H*′ = 1.3). The diversity at seamounts was highest at station SF7 (*H*′ = 2.5), followed by station SF5 (*H*′ = 2.2). The lowest diversity at seamounts was observed at station SFX (*H*′ = 1.3). The evenness values were similar between subsystems (*J*: 0.7 for SM and 0.8 for OI, mean; Wilcoxon test, W = 15, *P* = 0.594). The evenness for oceanic islands was highest at station ST17 (*J* = 1.0) followed by station ST22 (*J* = 0.9). The lowest evenness at oceanic island stations was observed at ST21 (*J* = 0.5). The evenness at seamounts was highest at stations SF7 and SF8 (*J* = 0.9). The lowest evenness at seamounts was observed at station SF9 (*J* = 0.5) (Table 2). Species accumulation curves combining trawl and ROV data for each subsystem (seamount and oceanic island) showed that a sufficient level of sampling was not attained based on the species accumulation curves not reaching asymptotes (Fig. S1).

**Table 2:** OTU richness based on trawl data, total *S* (trawl + ROV) data and diversity and evenness indexes obtained from relative OTU abundances of only trawl data.

Site	Subsystem	OTUs richness (*S*)	Total *S* (trawl + ROV)	Diversity (*H*´)	Evenness (*J*´)
ST17	OI	4	15	1.3	1.0
ST18	OI	–	10***	–	–
ST20	OI	27	35	2.5	0.8
ST21	OI	13	15	1.3	0.5
ST22	OI	6	11	1.6	0.9
Mean	OI	12	17	1.7	0.8
SF2	SM	–	14***	–	–
SF5	SM	32	–	2.2	0.6
SF6	SM	19	27	2.2	0.7
SF7	SM	19	35	2.5	0.9
SF8	SM	6	8	1.6	0.9
SF9	SM	45	50	1.9	0.5
SFX	SM	11	13	1.3	0.6
mean	SM	22	24	1.9	0.7

Subsystem OI = oceanic islands and SM = seamounts. Only ROV (***).

### Community comparisons

**Figure 5:**
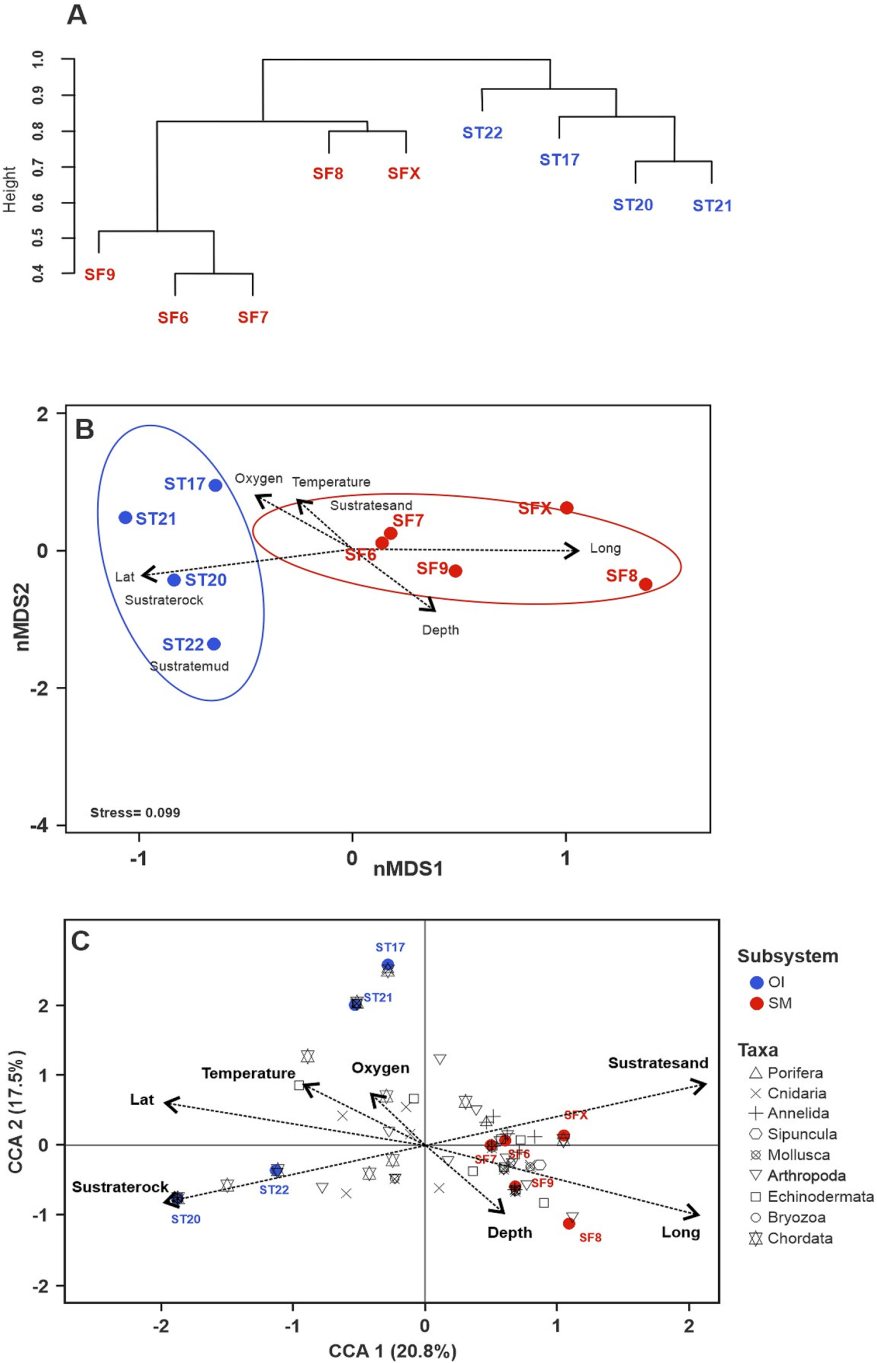
(**A**) Cluster analysis (UPGMA method) based on Bray–Curtis dissimilarity, (**B**) non-metric multidimensional scaling (nMDS) ordination plot based on presence/absence data of the benthic megafauna, and (**C**) canonical correspondence (CCA). Analysis based on the community structure of benthic megafauna, grouped at major taxa level, from stations sampled at Desventuradas Islands (blue markers) and seamounts (red markers) of the Nazca Desventuradas Marine Park. Vectors in (**B**) and (**C**) represent contribution of environmental descriptors (salinity, oxygen, temperature, substrate type, depth, latitude and longitude), and ellipses in (**B**) represent the 95% confidence interval. Image generated using R software (version 4.0.3)^47^.

The cluster (Fig. 5A) and the nMDS (Fig. 5B) analyses with combined data, revealed differences in the community structure between the stations, which clustered in two groups: (1) oceanic islands and (2) seamounts. This difference was corroborated by the PERMANOVA analysis based on combined data. Subsystem types (R^2^ = 0.273, *F* = 3.234, *p* < 0.001) and depth (R^2^ = 0.180, *F* = 2.132, *p* = 0.002) were significant variables in structuring the benthic communities of NDMP. The interaction between subsystem type and depth was not significant (R^2^ = 0.126, *F* = 1.497, *p* = 0.097) (Table S3).

Besides, when performed for each type of sampling alone (i.e., only trawl or only ROV data), a similar grouping pattern was observed in the cluster and nMDS analyses. However, while oceanic islands and seamounts formed separate groups for only trawl data (Fig. S2), one seamount, SF2, grouped with oceanic islands when analysed by only ROV data (Fig. S3). In both cases, PERMANOVA analysis corroborated differences for subsystems (Trawl: R^2^ = 0.155, F = 1.511, *p* = 0.002; ROV: R^2^ = 0.205, F = 2.662, *p* = 0.001) (Table S3). Depth was a significant factor for ROV data (R^2^ = 0.155, F = 2.018, *p* = 0.009) but not for trawl data (R^2^ = 0.118, F = 1.146, *p* = 0.171). No significant interaction was observed between factors for either trawl or ROV data (Trawl: R^2^ = 0.126, F = 1.497, *p* = 0.339; ROV: R^2^ = 0.102, F = 1.333, *p* = 0.196) (Table S3).

The first two axes of the CCA represented ~ 38% of the total variance and two groups of stations were evident (Fig. 5C). The position and spacing of stations along CCA axis 1 shows that stations with negative scores were oceanic islands which positively correlated with increasing latitude, temperature, oxygen and rocky habitat. In contrast, the seamounts were grouped on the positive axis 1 and correlated with increasing depth, longitude and coarse sand habitats. Stations did not clearly distribute along CCA axis 2, for which oxygen, depth and substrate type were the primary contributors among the environmental data. Overlaying faunal contributions revealed Chordata were mainly associated with Desventuradas islands stations, whereas invertebrates such as Arthropoda, Echinodermata and Mollusca were associated with seamounts along the positive axis 1 of the CCA. A large part of the invertebrates (mollusks: 25 OTUs, crustaceans: 18 OTUs and echinoderms: 8 OTUs), in particular, the gastropods *Chryseofusus kazdailisi*, *Calliostoma* sp., *Atrimitra isolata*, *Cryptogemma praesignis*, the crustaceans *Munida diritas*, *Heteronida* sp., *Paragiopagurus* spp., *Ebalia sculpta*, and the irregular urchins *Brissopsis* sp. and *Spatangus* sp., were exclusively associated with seamounts F6, SF7 and SF9. Conversely, most of the fishes, including *Tetronarce* sp., *Callyonimus* sp., *Parapercis* cf. *dockinsi, Seriola lalandi*, *Nemadactylus gayi*, and *Gonorynchus greyi*, and a small portion of invertebrates (mollusks: 2 OTUs, crustaceans: 6 OTUs and echinoderms: 5 OTUs), including the bivalve *Arca* cf. *fernanandezensis,* stomatopod *Hemisquilla ensigera,* snapping shrimp *Alpheus* cf. *romensky,* dollars *Clypeaster* spp. and the sea star *Astrostole platei*, were associated with stations around the islands (ST20 and ST21).

**Figure 6:**
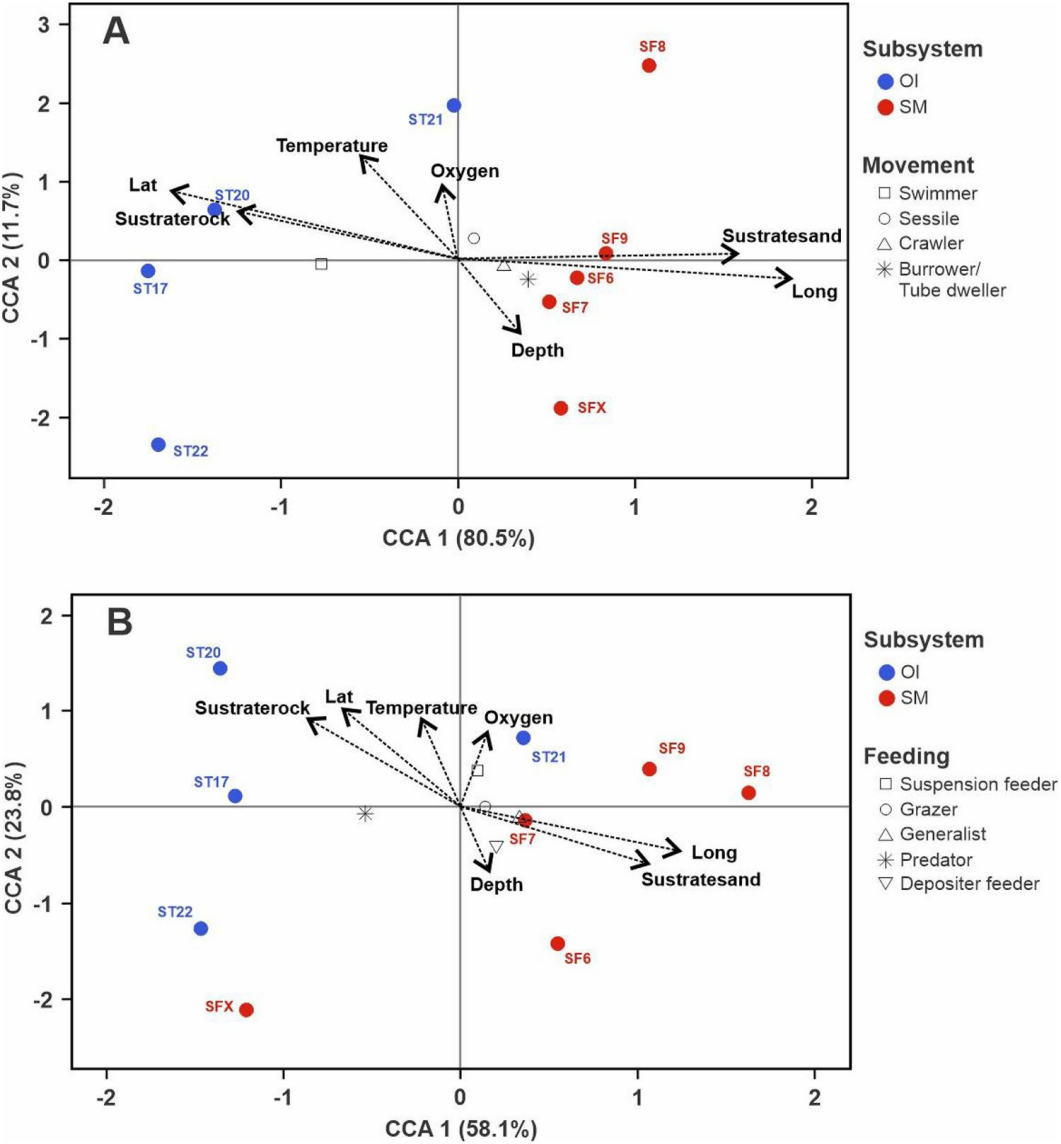
Canonical correspondence analyses (CCA) analysis based on the community structure of benthic megafauna grouped by movement type (**A**) and feeding mode (**B**) from stations sampled at Desventuradas Islands (OI, blue markers) and seamounts (SM, red markers) of the Nazca Desventuradas Marine Park. Vectors represent the contribution of environmental descriptors (oxygen, temperature, substrate type, depth, latitude and longitude). Image generated using R software (version 4.0.3)^47^.

Regarding functional traits of benthic communities, the first two axes of the CCA analysis, based on movement type (Fig. 6A) and feeding modes (Fig. 6B), accounted for 92.2% and 81.9% of the total variance, respectively. This analysis showed that oceanic islands were mainly dominated by mobile fauna and predators (e.g., the fishes *Squalus mitsukurii, Lotella* cf. *fernandeziana*, *Helicolenus lengerichi* and *Scorpaena thomsoni*, and large crustaceans (e.g., *Projasus bahamondei* and *Paromola rathbuni*), whereas seamounts farther from the islands were dominated by sessile and hemisessile invertebrates (i.e. crawlers and burrowers/tube dwellers) that were mainly suspension and deposit feeders (e.g., *Hormathia* sp., *Protoptilum* sp., *Paragiopagurus* spp.).

## Discussion

This study constitutes the most complete and updated description of benthic habitats and fauna for the upper slope of Desventuradas Islands and summits of nearby seamounts within the NDMP. ROV videos allowed the first description of benthic habitats of the Desventuradas Islands and their surrounding seamounts at a fine scale (meters to centimeters). Although we are aware of the limitations of the present study (e.g., only one ROV dive and/or trawl per site), we consider that our information presents a fundamental base-line knowledge of the benthic fauna and environment in this relatively understudied region of the Pacific Ocean, with a high value for conservation.

Of the three macrohabitats (Fig. 2, Table 1) observed in this study, relatively homogeneous coarse sand with patches of habitat-forming sessile species and rhodoliths was the most commonly observed at oceanic islands and seamounts. These observations are consistent with the description of the macrohabitat made by “Pristine Seas Expedition” at depths of 20 to 350 m^51^ at the Desventuradas Islands and reports of substrates on other seamounts of the SGR^15, 19^ as well as of the eastern Pacific Ocean^52^, northeast Atlantic^53^ and equatorial Atlantic^9^. For example, Parin et al.^15^ reported, based on collections and observations made from a manned submersible, that the upper slopes of SGR seamounts located > 400 km NW of Desventuradas Islands had a soft bottom composed of biogenic sands derived from shells of local mollusks with some pelagic pteropods, foraminiferal material and fossil corals. We observed a change in the kind of sediment/ bottom type with predominantly coarse sands at the 150–180 m stations and predominantly fine sands at 220–370 m. This pattern of sediment change with depth has also been recorded in other deep-sea areas^54–57^.

The rocky macrohabitat observed on oceanic island ST18 (Fig. 2A) is similar to other islands located in the southwest Pacific. For example, Lord Howe Island (~ 33°S, 159°E, ~ 600 km E of Australia) is also an isolated, endemism hotspot with several similarities to Desventuradas Islands^20^. The macrohabitat of Lord Howe Island at a depth of 50 m is characterized by having a rocky bottom covered with encrusting red algae and being dominated by urchins (*Tripneustes* and *Centrostephanus*)^20, 30^.

The rocky substrate (i.e., steep slope of compact cemented rock) observed at seamount SF2 (Fig. 2F) is consistent with the macrohabitat of other seamounts of the NR (e.g., Professor Mesyatzev, Soldatov, Ikhtiandr and Ekliptika; ~ 81–83°W) reported by Parin et al.^15^. This macrohabitat is characterized by the presence of a longitudinal trench in the cemented rock, which gives shelter to numerous species of fishes (e.g., *Helicolenus lengerichi* and *Scorpaena thomsoni*) and large crustaceans (e.g., the Chilean jagged lobster *Projasus bahamondei* and the Juan Fernández carrier crab *Paromola rathbuni*) (Fig. 2F).

Until recently, most seamount research related to benthic habitat heterogeneity and complexity has focused on larger-scale variations (macrohabitats)^58^. Unlike hard-bottomed habitats, soft-bottom habitats have generally been considered unstructured, homogeneous environments; consequently, these habitats have been studied primarily from a landscape perspective (usually at a kilometer scale)^58^. Hence, Clark et al.^59^ suggested that the concept of seamounts as a single, relatively well-defined habitat type appears outdated, giving way to a growing recognition that within seamount variability can have different spatial scales. Although not statistically tested, our results suggest a higher faunal diversity associated with biogenic microhabitats compared to sites with few or no biogenic microhabitats (e.g., SF8 and SFX), further supporting the importance of such small-scale differences in understanding the mechanisms involved in maintaining species diversity and the linkages between habitat-forming species and users of these habitats (Table S4). The presence of multiple distinct morphologies of the deep seabed, such as, scars, channels, mud, rocks and sand, together with habitat-forming species (e.g., sponge, anemones and stylasterids corals) provide an environment that is home to numerous species, resulting in increases in diversity^16, 60^. Besides, habitat-forming species are known to promote local complexity and heterogeneity of the seafloor at small (microhabitat) to large (macrohabitat) scales and to provide suitable refuge and nursery zone for smaller mobile benthic organisms^2, 24, 60, 61^. Consistent with this pattern, it appears that OTU richness may be higher where habitat-forming species, such as sea pens (*Protoptilum* and *Scleroptilum*), anemones (*Hormathia* sp.), tube-dwelling anemones and stylasterids corals were present (Table S4). Microhabitats within and among seamounts differed based on small modifications in the seafloor substrate and the presence of habitat-forming species such as anemones (*Hormathia* sp.), tube-dwelling anemones (cerianthids) and sea pens (*Protoptilum* sp. and *Scleroptilum* sp.). A marked pattern was observed between substrate type (soft vs. hard) and the type of habitat-forming species; anemones and sea pens of the genus *Protoptilum* were observed frequently on soft bottoms, whereas sea pen of the genus *Scleroptilum* and stylasterids corals were reported on hard bottoms. Similar patterns have been described for deeper habitats (> 400 m depth) of NR and SGR seamounts and oceanic islands^15, 19^ and also for New Caledonia, particularly on the Norfolk Ridge seamounts^16^.

Habitat-forming species were distributed as small patches that supported a higher concentration of fauna compared to the surrounding seafloor, providing a structure that can support more species than habitats without such complexity^16^. For example, at NDMP seamounts, squat lobsters (*Munida diritas* and *Phylladiorhynchus pusillus*), crabs (*Latreillia* sp.) and juveniles of *Caprodon longimanus* were observed hidden among anemones (Fig. 3D). This type of seamount microhabitat is mainly composed of coarse sand and rhodoliths with anemones, sea pens and tube-dwelling anemones, which likely provide nursery zones for some fishes, as suggested by ROV observations of the presence of juveniles of *C. longimanus* in these anemone patches (SF6 and SF7 seamounts). Another role of microhabitats is contributing to predator–prey dynamics; for example, the use of microhabitats not only plays a role in predator avoidance by providing shelter but can also play a role in ambush tactics by providing a focal site where the prey may not recognize the predator^28^. In our study, squat lobsters (*Munida diritas*) were observed hunting mysidaceans that foraged around anemone tentacles. In a recent description, Gallardo et al.^62^ mentioned that the white coloration of the squat lobster favors camouflage among coquina sediments. Similar observations have been reported by Auster et al.^28^, who documented some fauna (e.g., red hake, ocean pout, longhorn sculpin, squid, conger eel, and black-bellied rosefish) using microtopographic features and resources of microhabitat to block visual and acoustic (i.e., proprioceptive) recognition of occupants in ambush tactics.

The NR and SGR are considered biologically-unique because of their remarkably high endemism^11, 20^. Previous studies have found that the Nazca-Desventuradas region has unique marine ecosystems that consist of a mixture of tropical, subtropical and temperate species with a strong affinity with the Indo-Pacific^18, 20^. We report a total of 118 OTUs (131 if adding non-living specimens or shells), of which 50 invertebrates and 30 fishes were identifiable to species level (Table S2). The number of species reported was much higher than the review of Castilla^63^ that reported 50 species (23 invertebrates and 27 fishes) and the Chilean expeditions (i.e., CIMAR 6 and Pristine Sea Expedition) that reported a total of 37 species. This increase in species number for the region is, most probably, due to the few previous scientific studies that focused on benthic megafauna^64^. Although the sampling effort deployed in the area is still far from being satisfactory, as indicated by the species accumulation curves (Fig. S1), this study reports 43 genus-level OTUs not previously mentioned in the NDMP area (Table S2), of which only 14 were assigned to the level of species (new records for the area), with the remaining ones being potentially new species to science. New records from this study for the Nazca-Desventuradas Marine Park include: two genera of Pennatulaceans (*Protoptilum* and *Scleroptilum*); seven genera and five species of polychaetes (*Lanice sp.*, *Chloeia* sp., *Eunice decolorhami*, *Phyllodoce pseudopatagonica*, *Trypanosyllis* cf. *zebra* and *Mesochaetopterus minutus*), bringing the total to 13 genera and five species^15, 65, 66^; one genus of Sipuncula (*Aspidosiphon* sp.); 14 genera and four species of gastropods (*Iniforis* cf. *limitaris*, *Atrimitra isolata*, *Chryseofusus kazdailisi*, *Cryptogemma praesignis*, *Architectonica karsteni*), bringing the total to 40 genera and 31 species^15, 21, 43, 67^; 17 genera and 15 species of crustaceans (e.g., *Miersiella haswelli*, *Zarenkolambrus minutus*, *Z. epibranchialis*, *Heteronida* sp, *Munida diritas* and *Hispidolambrus mironovi*) (Table S2), bringing the total to 25 genera and 23 species^42, 44, 45^; and two genera of fishes (*Tetronarce* sp. and *Callionymus* sp.). Fishes is the best documented group in the NDMP area, including reports by Sepulveda^68^, Pequeño & Lamilla^40^, Dyer & Wesneat^39^ and National Geographic & Oceana^51^ around the Desventuradas Islands from 5 to 2,215 m depth and the summit of the Stockman guyot (station SFX) at 200 to 370 m depth.

Therefore, it is probable that a greater sampling effort (i.e., higher areal coverage and including flanks and bases of seamounts) will continue revealing substantial numbers of novelties for the region. Although rare invertebrate species and possibly new species were observed in the present study, none of the new records appear to be endemic to a particular seamount (Table S2). However, finer-scale studies may help to uncover diversity patterns of species within and among seamounts in this region. Furthermore, because of limited sampling across the southeast Pacific, available information is still insufficient to assess the high endemism and regional affinity of the NDMP fauna.

Although out of the scope of the present study, we provide records that would help to better assess the hypothesis of a faunal transition zone or "break" proposed by Mironov & Detinova^69^ and subsequently by Parin et al.^15^ and Mecho et al.^37^ which divides the hard-bottom seamounts of south NR from the soft bottom seamounts of the east SGR. Parin et al.^15^ suggested a faunistic break at ~ 88°W. Mecho et al.^70^ observed remarkable turnovers of echinoderms at ∼101° and ∼86°W of the SGR, where assemblages tended to differ more across seamounts, suggesting the effects of physical barriers to dispersion (e.g., currents) and habitat changes. Our data suggests a transition zone located at the intersection of SGR and NR (~ 82.5°W), as indicated by the separation among stations in the nMDS (Fig. 5B) and CCA (Fig. 5C) coinciding with the initial position (~ 82–84°W) proposed by Mironov & Detinova^69^. Differences among the communities can also be seen in the functional groups and composition of the fauna of the seamounts of the NDMP separated by this approximate longitude (Fig. S2). Seamount SF2 is the only hard-bottomed seamount explored, with a mobile fauna with a type of feeding associated with predators such as the spiny lobster *Projasus bahamondei* and shark *Squalus mitsukurii*. These characteristics are shared with other seamounts such as Zvezda to Bolshaya further northeast in the NR^15^. In contrast, the other seamounts studied coincide more with the characteristics reported for the SGR because they have a soft bottom and sessile/hemisessile benthic megafauna with a generalist feeding mode^15^.

The CCAs (Fig. 6) suggest that differences in feeding mode and mobility of the megafauna appear to be linked to habitat type, depth, temperature and oxygen. Such patterns have been observed in other seamounts or deep habitats reported in literature^15, 70^. The megafauna of the Desventuradas Islands was characterized by a comparable high frequency of occurrence and diversity of fishes (e.g., *Suezichthys rosenblatti*, *Paratrimma* sp., *Parapercis* cf. *dockinsi* and *Seriola lalandi*) and large predators such as the shark *Squalus mitsukurii*^7^*.* This predominance of fishes is likely the result of the islands having a biogenic structure that could support higher fish densities because of higher availability of nutrients, terrigenous contributions and eventual trophic subsidies by macroalgae (e.g., *Eisenia* cf. *cokeri*)^68, 71^. In contrast, the seamount fauna was characterized by having hemisessile and sessile fauna that are opportunistic suspension feeders (e.g., *Hormathia* sp. *Protoptilum* sp. *Scleroptilum*, and *Stylaster* sp.) or deposit feeders (e.g., *Paragiopagurus* spp. and *Munida diritas*). This pattern suggests that the seamounts of NDMP might have short food webs and low guild complexity, i.e., the majority of the animals in seamounts consume similar resources at a low trophic level^16, 23^. A similar pattern was described for the New Caledonian seamounts, where it has been hypothesized that the length of the food chain in seamounts is shorter than in other aquatic systems^16^. These authors also found that although the food web in seamounts is short, it is still relatively complex.

## Conclusions

This study constitutes a first descriptive addition to the knowledge of benthic micro- and macrohabitats of the slope of Desventuradas Islands and summits of nearby seamounts within the NDMP, providing the most complete description of their benthic megafauna to date. The benthic habitats of oceanic island slopes and seamounts within the NDMP are home to a large diversity of habitats, including rock, rhodoliths, sandy bottoms, silty bottoms, vertical walls and caves. Our study highlights that faunal composition differed with seafloor habitat. For example, higher values of diversity and OTU richness were observed in areas with a predominance of habitat-forming species, which points to the need to study seamounts at finer scales (centimeters to meters) to better understand the links of habitat-forming species and faunal communities and the potential effects that would result from the loss of macro- and microhabitat-forming species. Currently, these habitats within the NDMP are in an apparent pristine state (i.e., without evident signs of human impacts, such as marine litter or trawled areas). Thus, these seamounts, now under protection, provide a regional unique opportunity to monitor effects of climate change without the confounding factor of human activities, such as fishing, deep seabed mining or bottom trawl fishing, and could also be useful for comparisons with other places in the world where such closures have been implemented for seamounts in the Azores, SW Indian Ocean, New Zealand, Hawaiian Islands and Palau, to mention a few.

## Supplementary Information

Supplementary Information 1.

Supplementary Legends.

## References

[1] Yesson C, Clark MR, Taylor ML, Rogers AD (2011). The global distribution of seamounts based on 30 arc seconds bathymetry data. Deep. Res. Part I Oceanogr. Res. Pap..

[2] Preez CDu, Curtis JMR, Clarke ME (2016). The structure and distribution of benthic communities on a shallow seamount (Cobb Seamount, Northeast Pacific Ocean). PLoS ONE.

[3] Auster PJ (2011). Definition and detection of vulnerable marine ecosystems on the high seas: problems with the ‘move-on’ rule. ICES J. Mar. Sci..

[4] Watling L, Auster PJ (2017). Seamounts on the high seas should be managed as vulnerable marine ecosystems. Front. Mar. Sci..

[5] Cho, W. W. *Faunal Biogeography, Community Structure, and Genetic Connectivity of North Atlantic Seamounts* (Massachusetts Institute of Technology & Woods Hole Oceanographic Institution, 2008).

[6] Rogers, A. D. *The Biology of Seamounts: 25 Years on*. *Advances in Marine Biology* vol. 79 (Elsevie, 2018). 10.1016/bs.amb.2018.06.001 30012275

[7] Wagner, D. *et al.* The Salas y Gómez and Nazca ridges: a global diversity hotspot in need of protection. 28 (2020).

[8] Kvile KO, Taranto GH, Pitcher TJ, Morato T (2014). A global assessment of seamount ecosystems knowledge using an ecosystem evaluation framework. Biol. Conserv..

[9] Victorero L, Robert K, Robinson LF, Taylor ML, Huvenne VAI (2018). Species replacement dominates megabenthos beta diversity in a remote seamount setting. Sci. Rep..

[10] Yesson, C. *et al.* Improved bathymetry leads to 4000 new seamount predictions in the global ocean. *UCL Open Environ.* **Preprint**, 1–12 (2020). 10.14324/111.444/ucloe.000030 PMC10171409 37228795

[11] Gálvez Larach M (2009). Montes submarinos de Nazca y Salas y Gómez: una revisión para el manejo y conservación. Lat. Am. J. Aquat. Res..

[12] Jarrard RD, Clague DA (1977). Implications of Pacific Island and seamount ages for the origin of volcanic chains. Rev. Geophys..

[13] Chave EH, Jones AT (1991). Deep-water megafauna of the Kohala and Haleakala slopes, Alenuihaha Channel Hawaii. Deep Sea Res. Part A Oceanogr. Res. Pap..

[14] Kitchingman A, Lai S, Morato T, Pauly D, Pitcher TJ (2008). How many seamounts are there and where are they located?. Seamounts: Ecology, Fisheries & Conservation, Series 12.

[15] Parin, N. V., Mironov, A. N. & Nesis, K. M. *Biology of the Nazca and Sala y Gómez submarine ridges, an outpost of the Indo-West Pacific fauna in the eastern Pacific ocean: composition and distribution of the fauna, its communities and history*. *Advances in Marine Biology* vol. 32 (1997).

[16] Samadi S, Schlacher T, Richer de Forges B, Pitcher T (2007). Seamount benthos. Seamounts: Ecology, Fisheries and Conservation.

[17] Mironov, A. N., Molodtsova, T. N. & Parin., N. V. Soviet and Russian studies on seamount biology. (2006).

[18] Fernández M, Pappalardo P, Rodríguez-Ruiz MC, Castilla JC (2014). Síntesis del estado del conocimiento sobre la riqueza de especies de macroalgas, macroinvertebrados y peces en aguas costeras y oceánicas de Isla de Pascua e Isla Salas y Gómez. Lat. Am. J. Aquat. Res..

[19] Easton, E. E. *et al.* Chile and the Salas y Gómez Ridge. In *Mesophotic Coral Ecosystems* 477–490 (Springer, 2019). 10.1007/978-3-319-92735-0_27.

[20] Friedlander AM (2016). Marine biodiversity in Juan Fernández and Desventuradas islands, Chile: global endemism hotspots. PLoS ONE.

[21] Sellanes J, Salisbury RA, Tapia JM, Asorey CM (2019). A new species of *Atrimitra* Dall, 1918 (Gastropoda: Mitridae) from seamounts of the recently created Nazca-Desventuradas Marine Park Chile. PeerJ.

[22] Gaymer, C. F. *et al. Plan General de Administración y su Valoración Económica. Informe final proyecto FIPA 2016–31 ‘Bases técnicas para la gestión del Parque Marino Nazca-Desventuradas y propuesta de Plan General de Administración’* (2018).

[23] Clark MR (2010). The ecology of seamounts: structure, function, and human impacts. Ann. Rev. Mar. Sci..

[24] Henry LA (2014). Environmental variability and biodiversity of megabenthos on the Hebrides Terrace Seamount (Northeast Atlantic). Sci. Rep..

[25] Jones CG, Lawton JH, Shachak M (1994). Organisms as ecosystem engineers. Oikos.

[26] Morgan NB, Goode S, Roark EB, Baco AR (2019). Fine scale assemblage structure of benthic invertebrate megafauna on the North Pacific Seamount Mokumanamana. Front. Mar. Sci..

[27] Davies JS (2015). Benthic assemblages of the Anton Dohrn Seamount (NE Atlantic): defining deep-sea biotopes to support habitat mapping and management efforts with a focus on vulnerable marine ecosystems. PLoS ONE.

[28] Auster PJ, Malatesta RJ, Larosa SC (1995). Patterns of microhabitat utilization by mobile megafauna on the southern New England (USA) continental shelf and slope. Mar. Ecol. Prog. Ser..

[29] Uzmann JR, Cooper RA, Theroux RB, Wigley RL (1977). Synoptic comparison of three sampling techniques for estimating abundance and distribution of selected megafauna: submersible vs. camera sled vs. otter trawl. Mar. Fish. Rev..

[30] Valentine JP, Edgar GJ (2010). Impacts of a population outbreak of the urchin *Tripneustes gratilla* amongst Lord Howe Island coral communities. Coral Reefs.

[31] Greene H (1999). A classification scheme for deep seafloor habitats. Oceanol. Acta.

[32] Greene H, O’Connell V, Brylinsky C, Reynolds J, Reynolds J, Greene HG (2008). Marine Benthic Habitat classification: What’s Best for Alaska?. Marine Habitat Mapping Technology for Alaska.

[33] Naar, D. F., Johnson, K. P., Wessel, D., Duncan, P. & Mahoney, J. Rapa Nui. 2001: Cruise report for Leg 6 of the Drift expedition aboard the R/V Revelle (2001).

[34] Haase KM, Stoffers P, Garbe-Schönberg CD (1997). The petrogenetic evolution of lavas from Easter Island and neighbouring seamounts, near-ridge hotspot volcanoes in the SE pacific. J. Petrol..

[35] Woods MT, Okal EA (1994). The structure of the Nazca Ridge and Sala y Gomez seamount chain from the dispersion of Rayleigh waves. Geophys. J. Int..

[36] Rodrigo, C., Foucher, N., Philippi, N. & Lara, L. E. Morfoestructuras volcánicas y sedimentarias de los montes submarinos de la región de las islas Desventuradas, basadas en el análisis de datos acústicos. 110–115 (2017).

[37] Mecho A (2021). Environmental drivers of mesophotic echinoderm assemblages of the Southeastern Pacific Ocean. Front Mar. Sci..

[38] VLC media player - Open Source Multimedia Framework and Player.

[39] Dyer BS, Westneat MW (2010). Taxonomía y biogeografía de los peces costeros del Archipiélago de Juan Fernández y de las islas Desventuradas Chile. Rev. Biol. Mar. Oceanogr..

[40] Pequeño G, Lamilla J (2000). The Littoral Fish Assemblage of the Desventuradas Islands (Chile) Has Zoogeographical Affinities with the Western Pacific. Glob. Ecol. Biogeogr..

[41] Raines, B. & Huber, M. *Biodiversity Quadrupled-Revision of Easter Island and Salas y Gómez Bivalves*. *Zootaxa* **106** (2012).

[42] Retamal MA, Moyano HI (2010). Zoogeografía de los crustáceos decápodos chilenos marinos y dulceacuícolas. Lat. Am. J. Aquat. Res..

[43] Sysoev, A. B. Gastropods of the family Turridae (Gastropoda:Toxoglosa) of the Nasca and Sala y Gómez underwater ridges. **124**, 245–260 (1990).

[44] Zarenkov NA (1969). Crabs of the familiy Leucosiidae (subfamilies Ebalinae an Iliinae) collected in tropical water of Indian and Pacific oceans waters of Indian and Pacific oceans. Bol. Nauk..

[45] Zarenkov NA (1990). Decapods (Stenopodidea, Brachyura, Anomura) of the underwater Nazca and Salas y Gómez Ridges. Tr. Instituta Okeanol. AN USSR.

[46] Barriga E, Salazar C, Palacios J, Romero M, Rodriguez A (2009). Distribucion, abundancia y estructura poblacional del langostino rojo de profundidad Haliporoides diomedeae (Crustacea: Decapoda: Solenoceridae). Lat. Am. J. Aquat. Res..

[47] R Core Team. R Core Team (2020). R: A language and environment for statistical computing. version 4.0.3. R Foundation for Statistical Computing, Vienna, Austria. https://www.R-project.org/ (2019).

[48] Oksanen J *et al.* vegan: Community Ecology Package.R package version 2.5-7. https://cran.r-project.org/package=vegan (2020).

[49] Jones D, Frid CLJ (2009). Altering intertidal sediment topography: effects on biodiversity and ecosystem functioning. Mar. Ecol..

[50] Wickham H (2009). ggplot2: Elegant Graphics for Data Analysis.

[51] National Geographic & Oceana. Islas Desventuradas. Biodiversidad marina y propuesta de conservación. 58 (2013).

[52] Levin LA, Nittrouer CA, Keating B, Fryer P, Batiza R, Boehlert G (1987). Textural characteristics of sediment on deep seamounts in the eastern Pacific Ocean between 10°N and 30°N. Seamounts, Islands and Atolls, 43.

[53] Lourido A, Parra S, Serrano A (2019). Preliminary Results on the Composition and Structure of Soft-Bottom Macrobenthic Communities of a Seamount: the Galicia Bank (NE Atlantic Ocean). Thalassas.

[54] Flach E, Muthumbi A, Heip C (2002). Meiofauna and macrofauna community structure in relation to sediment composition at the iberian margin compared to the goban spur (NE atlantic). Prog. Oceanogr..

[55] Levin LA, Gooday A, Tyler P (2003). The deep Atlantic Ocean floor. Ecosystems of the Deep Oceans.

[56] Thistle D, Tyler PA (2003). The deep-sea floor: an overview. Ecosystems of the World, Ecosystems of the Deep Sea.

[57] Louzao M (2010). Historical macrobenthic community assemblages in the Avilés Canyon, N Iberian Shelf: Baseline biodiversity information for a marine protected area. J. Mar. Syst..

[58] Kon K, Tsuchiya Y, Sato T, Shinagawa H, Yamada Y (2015). Role of microhabitat heterogeneity in benthic faunal communities in sandy bottom sediments of Oura Bay, Shimoda Japan. Reg. Stud. Mar. Sci..

[59] Clark MR, Schlacher TA, Rowden AA, Stocks KI, Consalvey M (2012). Science priorities for Seamounts: research links to conservation and management. PLoS ONE.

[60] Zeppilli D, Pusceddu A, Trincardi F, Danovaro R (2016). Seafloor heterogeneity influences the biodiversity-ecosystem functioning relationships in the deep sea. Sci. Rep..

[61] de la Torriente A (2019). Benthic habitat modelling and mapping as a conservation tool for marine protected areas: a seamount in the western Mediterranean. Aquat. Conserv. Mar. Freshw. Ecosyst..

[62] Gallardo M, Macpherson E, Tapia-Guerra JM, Asorey CM, Sellanes J (2021). A new species of *Munida* Leach, 1820 (Crustacea: Decapoda: Anomura: Munididae) from seamounts of the Nazca-Desventuradas Marine Park. PeerJ.

[63] Castilla, J. C. *Islas oceánicas chilenas: conocimiento científico y necesidades de investigación* (Ediciones Universidad Católica de Chile, 1987).

[64] Bahamonde, N. San Félix y San Ambrosio, las islas llamadas Desventuradas 85–99 (1987).

[65] Díaz-Díaz, O., Bone, D., Rodríguez, C. T. & Delgado-Blas, V. H. Poliquetos de Sudamérica. **Especial d**, 149 (2017).

[66] Díaz-Díaz OF, Rozbaczylo N, Sellanes J, Tapia-Guerra JM (2020). A new species of *Eunice* Cuvier, 1817 (Polychaeta: Eunicidae) from the slope of the Desventuradas Islands and seamounts of the Nazca Ridge, southeastern Pacific Ocean. A New Species Cuscus.

[67] Kantor Y, Sysoev A (1992). Latiaxis (Babelomurex) naskensis, a new species of Coralliophilidae (Gastropoda) from South-Eastern Pacific. Ruthenica.

[68] Sepulveda, J. I. Peces de las Islas Oceánicas Chilenas. In *Islas Oceánicas Chilenas: Conocimiento científico y necesidades de Investigaciones.* (ed. Castilla, J.) 225–246 (Ediciones Universidad Católica de Chile, 1987).

[69] Mironov, A. & Detinova., N. Bottom fauna of the Nazca and Sala y Gomez ridges. *Plankton and benthos from the Nazca and Sala y Gomez Submarine Ridges* 269–278 (1990).

[70] Lundsten L (2009). Benthic invertebrate communities on three seamounts off southern and central California USA. Mar. Ecol. Prog. Ser..

[71] Rex MA (2006). Global bathymetric patterns of standing stock and body size in the deep-sea benthos. Mar. Ecol. Prog. Ser..

[72] QGIS.org. QGIS Geographic Information System.QGIS Association. Version 3.10. https://www.qgis.org (2020).

