# Supplementary material for: First description of deep benthic habitats and communities of oceanic islands and seamounts of the Nazca Desventuradas Marine Park, Chile
Source: Sci Rep. 2021 Mar 18;11:6209. doi: 10.1038/s41598-021-85516-8 (PMC7973752; doi:10.1038/s41598-021-85516-8)
Supplement: Supplementary file 1 — Supplementary Information 1. [file 41598_2021_85516_MOESM1_ESM.pdf]

Table S1. Date, depth and positions of Agassiz trawl and ROV video transects conducted on the summits of seamounts and off Desventuradas islands between 22 October and 1 November, 2016. Subsystem OI = oceanic island (upper slope) and SM = seamount (top of seamount). Bottom time for both trawl and ROV are estimated from first to last bottom contact. Swept area is estimated just for trawls.

Station	Subsystem	Sampling method	Date	Depth range (m)	Latitude (initial-bottom)	Longitude (initial-bottom)	Bottom time (min)	Estimated swept area (m^2)
ST17	OI	Agassiz	2016-10-23	133-133	-26.26	-80.09	4.8	500
		ROV	2016-10-23	135-140	-26.26	-80.10	44	-
ST18	OI	Agassiz	NA	NA	NA	NA	NA	NA
		ROV	2016-10-23	43-50	-26.30	-80.06	68	-
ST20	OI	Agassiz	2016-10-22	135-145	-26.38	-79.89	4.8	300
		ROV	2016-10-22	128-130	-26.32	-79.88	54	-
ST21	OI	Agassiz	2016-10-22	150-150	-26.34	-79.89	10.2	900
		ROV	2016-10-22	149-150	-26.37	-79.88	56	-
ST22	OI	Agassiz	2016-10-23	220-340	-26.33	-80.09	15	1400
		ROV	2016-10-23	350-370	-26.33	-80.01	43	-
SF2	SM	Agassiz	NA	NA	NA	NA	NA	NA
		ROV	2016-10-31	280-305	-24.74	-82.52	71	-
SF5	SM	Agassiz	2016-11-01	172- 180	-25.33	-82.88	7.2	500
		ROV	NA	NA	NA	NA	NA	NA
SF6	SM	Agassiz	2016-10-28	172-176	-25.55	-82.39	13.2	1200
		ROV	2016-10-28	150-176	-25.55	-82.39	78	-
SF7	SM	Agassiz	2016-10-28	176-176	-25.65	-82.47	10.2	900
		ROV	2016-10-28	170-174	-25.65	-82.47	53	-
SF8	SM	Agassiz	2016-10-27	199-282	-25.71	-83.31	10.2	900
		ROV	2016-10-27	220-250	-25.71	-83.01	44	-
SF9	SM	Agassiz	2016-10-27	204-215	-25.77	-83.16	15	1400
		ROV	2016-10-27	200-203	-25.77	-83.31	71	-
SFX	SM	Agassiz	2016-10-31	225-230	-25.08	-82.00	15	1400
		ROV	2016-10-31	220-226	-25.08	-82.00	43	-

Table S2. Checklist of benthic megafauna of oceanic islands and seamounts of Nazca-Desventuradas Marine Park with taxonomic classification and station presence and depth observations. New records for the area indicated with an asterisk (*) and shell collections (i.e., non-living specimens, not included in the analysis) are indicated with a triangle (▲). (Last taxonomic revision on 25 Nov 2020).

Phylum	Class	Order	Family	Taxa	Station	Depth range (m)
Porifera	Demospongiae	Undetermined	Undetermined	Porifera und. sp.1	S21, SF5, SF6, SF7, SF9, SFX	150-230
		Undetermined	Undetermined	Porifera und. sp.2	ST18	50
		Undetermined	Undetermined	Porifera und. sp.3	S17	140
Cnidaria	Anthozoa (Subclass: Hexacorallia)	Scleractinia	Caryophyllidae	*Caryophyllia* sp.	ST20, SF5, SF7, SF8, SF9	150-200
				Desmophyllum sp.	ST20	150
			Dentdrophylliidae	*Dendrophyllia* sp	SF9	200
		Anthipatharia	Anthipathidae	*Stichopathes* sp.	SF9	200
		Actinaria	Hormathiidae	*Calliactis* sp.	SF7, SF9	150-200
				Hormathiidae und.	ST20, ST21, SF5, SF6, SF7, SF9	150-200
	Anthozoa (Subclass: Octacorallia)	Pennatulacea	Protoptilidae	*Protoptilum* sp.*	ST20, SF6, SF7	150-176
			Scleroptilidae	*Scleroptilum* sp.*	ST20, ST21, SF2, SF7	150-280
		Alcyonacea	Plexauridae	*Swiftia* sp.	ST17	140
	Anthozoa (Subclass Ceriantharia)	Undetermined	Undetermined	Ceriantharia und.	SF6, SF7, SF9, SFX	176-230
	Hydrozoa	Leptothecata	Plumulariidae	*Plumularia* sp.1	ST20	150
				Plumularia sp.2	ST20, SF9	150-200
			Sertularellidae	*Sertularella* sp.	SF9	200
		Anthoathecata	Stylasteridae	*Stylaster* cf. *marenzelleri*	ST18, ST20, SF2	150
Annellida	Polychaeta	Amphinomida	Amphinomidae	*Chloeia* sp.*	SF6, SFX	176-230

Phylum	Class	Order	Family	Taxa	Station	Depth range a (m)
		Eunicida	Eunicidae	*Eunice decolorhami**	SF5, SF6, SF7, SF9, SFX	150-230
		Phyllodocida	Phyllodocidae	*Phyllodoce pseudopatagonica* *	ST21, SF6, SF7, SF9, SFX	150-230
			Syllidae	*Syllis* sp.*	SF9	200
				Trypanosyllis cf. *zebra* *	SF6	176
				Trypanosyllis sp.*	SF9	200
		Sabellida	Serpulidae	*Serpula* sp. *	ST20, SF5	150-180
				Spirorbis sp.	ST20, SF5	150-180
		Terebellida	Ampharetidae	Ampharetidae und.	SF9	200
			Terebellidae	*Lanice* sp.	ST17, SF2, SF5, SF6, SF7, SF9, SFX	150-280
		-	Chaetopteridae	*Mesochaetopterus minutus* *	SF9	200
Sipuncula	Phascolosomatidea	Aspidosiphonida	Aspidosiphonidae	*Aspidosiphon* sp. *	SF5, SF9, SFX	180-230
Mollusca	Bivalvia	-	Lyonsiidae	*Entodesma cuneata*	SF5	180
		Cardiida	Cardiidae	*Frigidocardium thaanumi* ▲	SF5, SF7, SF8	150-282
		Arcida	Glycymerididae	*Tucetona kauaia*▲	ST17, ST20, SF5, SF7, SF9	150-200
			Arcidae	*Arca* cf. *fernandezensis*	ST21	150-180
		Venerida	Veneridae	*Timoclea keegani*▲	SF5, SF6, SF7, SF8, SF9	150-282
			Glossidae	*Meiocardia hawaiana*	SF5, SF9	180-200
		Pectinida	Pectinidae	*Cryptopecten bullatus*	SF5, SF7, SF9	150-200
		Mytilida	Mytilidae	*Gregariella* sp.	SF9	200

Phylum	Class	Order	Family	Taxa	Station	Depth range (m)
	Gastropoda	-	Triphoridae	*Iniforis* cf. *limitaris*▲	SF9	200
		-	Epitoniidae	*Epitonium* sp.▲	SF5, SF7	150-180
		Littorinimorpha	Capulidae	*Capulus* sp.	SF5, SF7, SF9	150-200
			Naticidae	*Natica* sp.	SF5	180
		Neogastropoda	Muricidae	*Latiaxis naskensis*	SF5	180
				Coralliophila sp.▲	ST20	180
				Orania sp.▲	SF9	200
			Terebridae	*Terebra* sp.▲	SF7, SF9	176-200
			Mitridae	*Atrimitra isolata**	SF2, SF5, SF6, SF9	150-200
			Mitromorphidae	*Mitromorpha maculata*▲	SF5	180
			Fasciolariidae	*Chryseofusus kazdailisi*	SF2, SF5, SF6, SF7, SF8, SF9, SFX	180
				Latirus sp.▲	SF9	200
			Turridae	*Cryptogemma praesignis*▲	SF2, SF5, SF7, SF9, SFX	150-280
			Costellariidae	*Vexillum* sp.▲*	SF9	200
		-	Architectonicidae	*Architectonica karsteni*	SF5, SF6, SF9	176-200
		Trochida	Calliostomatidae	*Calliostoma* sp.▲	SF5, SF7	180-282
		Cephalaspidea	Cylichnidae	*Cylichna* sp.▲	SF9	200
		Pteropoda	Cavoliniidae	*Cuvierina pacifica*▲	SF5, SF6, SF7, SFX	150-280
				Cavolinia tridentata▲	SF5, SF6, SF7, SF9, SFX	150-280
			Limacinidae	*Limacina rangii*▲	SFX	280

Phylum	Class	Order	Family	Taxa	Station	Depth range (m)
Arthropoda	Hexanauplia	Sessilia	Verrucidae	*Verruca scrippsae*	SF5, SF8	282
			Archaeobalanidae	*Solidobalanus nascanus*	SF5, SF6, SF9	176-200
		Lepadiformes	Poecilasmatidae	*Poecilasma crassa*	SF2, SF5, SF6, SF9	176-200
	Malacostraca (Superorder:Peracarida)	Isopoda	Corallanidae	*Argathona* sp.	SF5	180
		Amphipoda	Phrosinidae	*Phrosina* sp.	SF6, SF7, SF9, ST17	150-200
	Malacostraca (Subclass:Hoplocarida)	Stomatopoda	Hemisquillidae	*Hemisquilla ensigera*	ST21	150
	Malacostraca (Superorder: Eucarida)	Euphausiacea	Euphausiidae	*Thysanopoda* sp.	SFX	230
		Decapoda (Suborder: Dendrobranchiata)	Solenoceridae	*Hadropenaeus lucasii*	SF9	200
		Decapoda (Infraorder: Achelata)	Palinuridae	*Projasus bahamondei*	ST22, SF2	280-340
		Decapoda (Infraorder: Caridea)	Alpheidae	*Alpheus* cf. *romensky*	ST21	150
			Pandalidae	*Pleosinika* aff. *Serratifrons*	SF8	230
		Decapoda (Infraorder: Anomura)	Parapaguridae	*Paragiopagurus boletifer*	SF2, SF5, SF6, SF7, SF9	150-280
				Paragiopagurus ruticheles	SF5, SF7, SF8, SF9	180-282
			Galatheidae	*Phylladiorhynchus pusillus*	SF6, SF9	176-200
			Munididae	Munida *diritas**	SF6, SF7, SF9	150-200
				Heteronida sp.*	SF9	200
		Decapoda (Infraorder: Brachyura)	Homolidae	*Paromola rathbuni*	ST22, SF2, SF6, SF7, SF9	150-340

Phylum	Class	Order	Family	Taxa	Station	Depth range (m)
			Latreilliidae	*Latreillia pennifera* *	ST20, SF7, SF9	150-200
			Leucosiidae	*Ancylodactyla* cf. *nana**	ST17, SF7	150
				Ebalia sculpta	SF9	200
			Majidae	*Ageitomaia baeckstroemi*	ST20, ST22, SF9	150-340
				Majidae und. *	SF6, SF7	150-176
			Parthenopidae	*Hispidolambrus mironovi**	SF6	176
				Zarenkolambrus epibranchialis	SF7, SF9	150-200
				Zarenkolambrus minutus	SF9	200
			Xanthidae	*Miersiella haswelli** (morph.1)	SF6, SF7, SF9	150-200
				*Miersiella haswelli** (morph.2)	SF9	200
Echinodermata	Echinoidea	Spatangoida	Spatangidae	*Spatangus* sp.	SF6, SF7	150-176
			Palaeotropidae	*Scrippsechinus fisheri*	ST17, ST20, ST21, SF5, SF6, SF7, SFX	150-282
			Brissidae	*Brissopsis* sp.	SF5	180
		Clypeasteroida	Clypeasteridae	*Clypeaster isolatus*	ST17 ST20, ST21	140-150
				Clypeaster cf. *europacificus*	ST21	150
		Cidaroida	Cidaridae	Stereocidaris nascaensis	SF5, SF6, SF7, SF8, SF9	150-340
		Salenioida	Saleniidae	*Bathysalenia scrippsae*	SF8, SF9	200-282
		Diadematoida	Diadematidae	*Centrostephanus sylviae*	ST18, ST20	50-150
		Camarodonta	Trigonocidaridae	*Trigonocidaris albida*	SF8	200

Phylum	Class	Order	Family	Taxa	Station	Depth range (m)
	Asteroidea	Paxillosida	Peudarchasteridae	*Pseudarchaster* sp.	SF5, SF6, SF7, SFX	150-230
		Valvatida	Ophidiasteridae	*Tamaria* sp.	SF5, SF6	176-180
			Asterinidae	*Parvulastra* sp.	ST18, SF9	50-200
			Goniasteridae	*Anthenoides* sp.	SF7, SF9	176-200
		Forcipulatida	Asteriidae	*Astrostole platei*	ST18	50
	Ophiuroidea	Amphilepidida	Ophionereididae	*Ophionereis* cf. *perplexa*	ST20	150
Bryozoa	Gymnolaemata	Cheilostomatida	Cellariidae	*Cellaria* sp.	ST20	150
Chordata (Subphylum:Vertebrata)	Elasmobranchii	Torpediniformes	Torpedinidae	*Tetronarce* sp.	ST20	150
		Squaliformes	Squalidae	*Squalus mitsukurii*	ST17, ST22, SF9	140-340
	Actinopterygii	Scorpaeniformes	Triglidae	*Pterygotrigla picta*	ST20, ST22, SF5, SF8	176-340
				Triglidae und.	SF7	150
			Neosebastidae	*Maxilicosta reticulata*	ST22	340
			Scorpaenidae	*Scorpaena thomsoni*	ST20, SF2	150-280
			Sebastidae	*Helicolenus longerichi*	ST22, SF2	280-340
		Anguilliformes	Congridae	*Gnathophis* sp.	ST17, SF5	140-180
			Muraenidae	*Gymnothorax porphyreus*	ST18	50
		Pleuronectiformes	Bothidae	*Arnoglossus coeruleosticta*	ST20, ST22	150-340
			Paralichthyidae	*Paralichthys fernandezianus*	ST17	140
		Beryciforme	Monocentridae	*Monocentris reedi*	ST17, ST20, ST21	140-150

Phylum	Class	Order	Family	Taxa	Station	Depth range (m)
		Perciformes	Draconettidae	*Centrodraco striatus*	ST22, SFX	150-340
			Labridae	*Suezichthys rosenblatti*	ST18, ST20	50-150
				Pseudolabrus gayi	ST18	50
			Callanthiidae	*Callianthias parini*	ST20, SF2	150-280
				Callianthias platei	ST20	150
			Gobiidae	*Pascua caudilinea*	ST20	150
				Paratrimma sp.	ST20	150
			Callionymidae	*Callionymus* sp. *	ST20	150
			Serranidae	*Caprodon longimanus*	ST20, SF2, SF7	150-280
			Pinguipedidae	*Parapercis* cf. *dockinsi*	ST20	150
			Carangidae	*Seriola lalandi*	ST20	150
			Cheilodactylidae	*Nemadactylus gayi*	ST20	150
			Pomacentridae	*Chromis meridiana*	ST17	140
		Gadiformes	Moridae	*Lotella* cf. *fernandeziana*	ST17	140-280
		Myctophiformes	Myctophidae	*Diaphus* sp.	SFX	230
				Lampanyctus cf. *intricarius*	SFX	230
		Sygnathiformes	Centriscidae	*Macroramphosus scolopax*	ST20	150
				Notopogon fernandezianus	ST17, SF7, SF9	140-200
		Gonorhynchiformes	Gonorynchidae	*Gonorynchus greyi*	ST17	140

Table S3. Summary of PERMANOVA (10,000 runs) of species composition for each type of sampling (trawl and ROV) and combined data (Total S), based in Bray-Curtis similarity index, in function of subsystem, depth and interaction.

**Type of sampling**	**Factor**	**MSS**	**R^2**	**F**	***P*-value**
ROV	Subsystem	0.6638	0.2046	2.6621	0.0012**
	Depth	0.5031	0.1550	2.0175	0.0097**
	Habitat: depth	0.3325	0.1025	1.3333	0.1963
Trawl	Subsystem	0.6332	0.1553	1.5106	0.0019**
	Depth	0.4805	0.1178	1.1463	0.1710
	Habitat: depth	0.4491	0.1101	1.0714	0.3391
Total S	Subsystem	0.71264	0.27257	3.2336	0.00020***
	Depth	0.46991	0.17973	2.1322	0.00160**
	Habitat: depth	0.33002	0.12623	1.4975	0.09739

Table S4. Number of Habitats forming species and number of the topographic elements per site.

Sites	N° Habitats forming-species	N° Topographic elements	Total
ST17	2	1	3
ST18	3	2	5
ST20	7	1	8
ST21	5	2	7
ST22	0	1	1
SF2	2	3	5
SF5	4	-	4
SF6	4	4	8
SF7	6	4	10
SF8	2	2	4
SF9	6	4	10
SFX	2	2	4

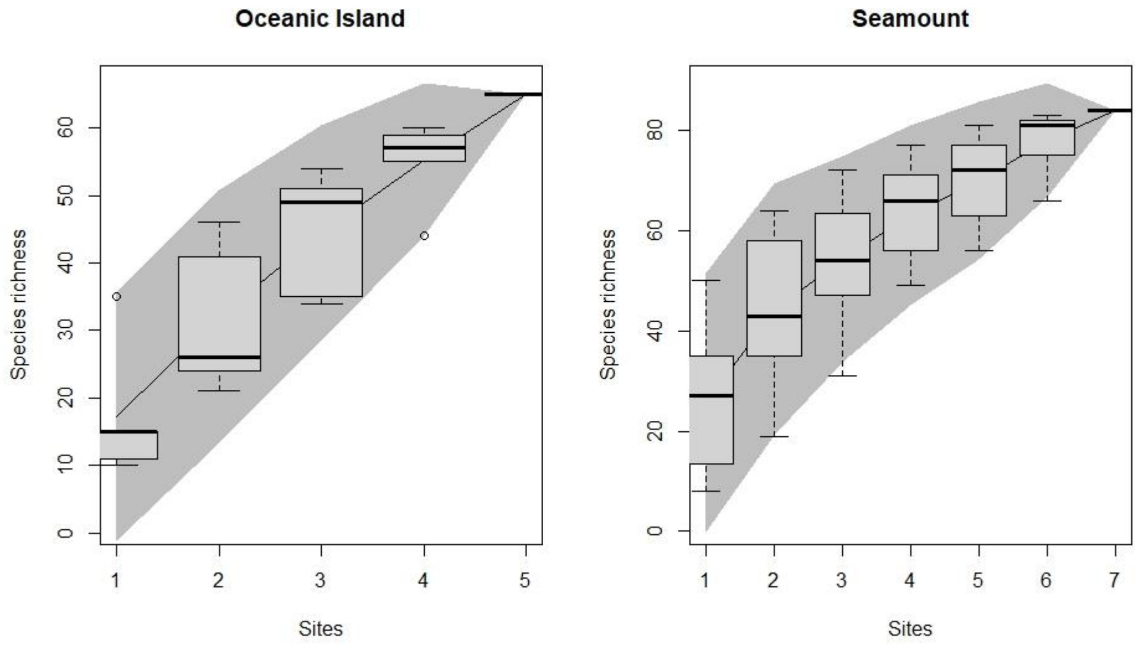

Fig. S1. Species accumulation curves for the stations sampled within the Nazca Desventuradas Marine Park. OTU richness (*S*) of each subsystem was estimated based on integrated (total S) presence/absence date. Box and whisker plots represent the 95% confidence interval.

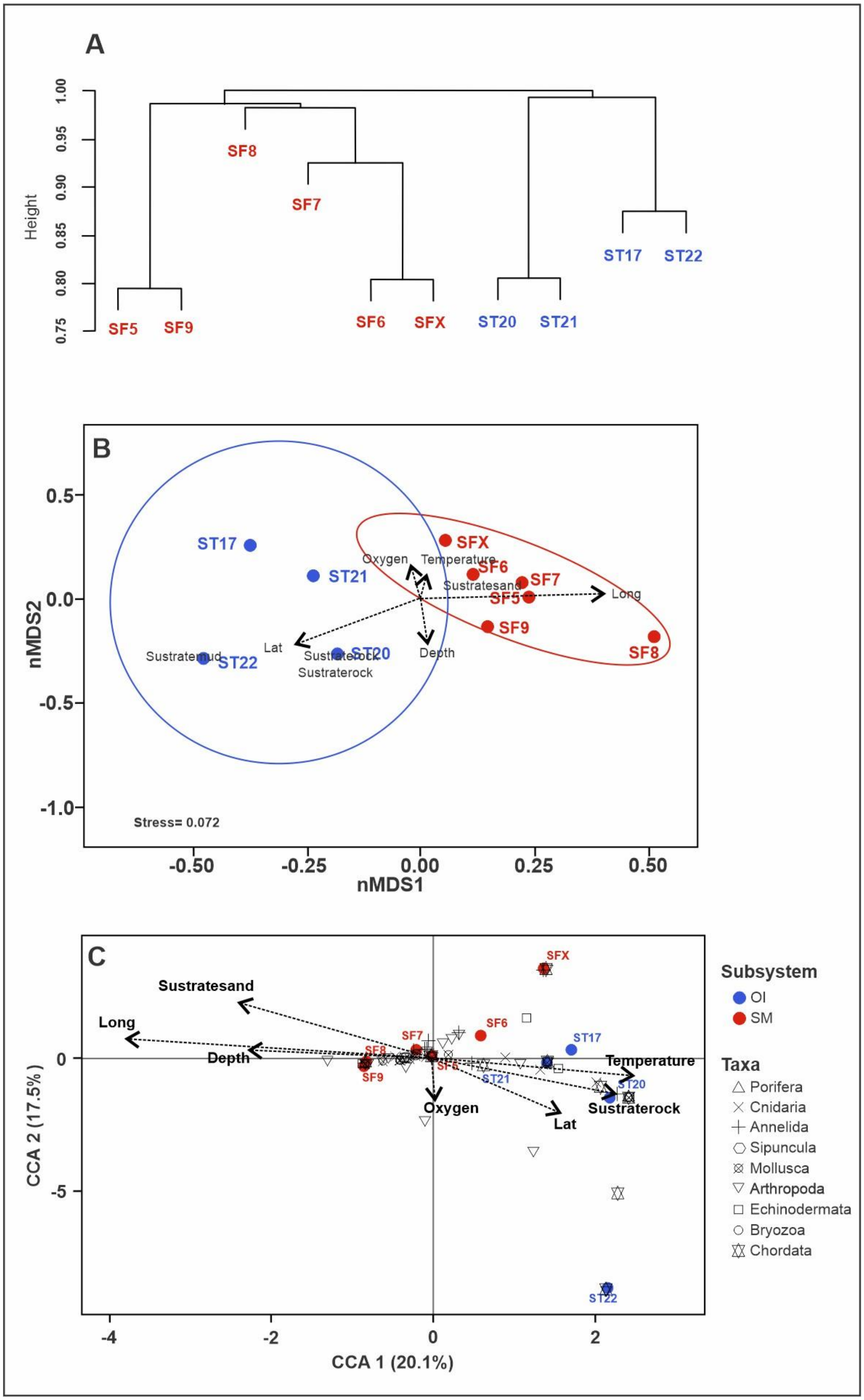
A
Height
1.00
0.95
0.90
0.85
0.80
0.75
SF8
SF7
SF5
SF9
SF6
SFX
ST20
ST21
ST17
ST22
B
nMDS2
nMDS1
Oxygen
Temperature
Sustratesand
Long
Lat
Depth
Sustratemud
Sustraterock
Stress= 0.072
C
Sustratesand
Long
Depth
Oxygen
Lat
Temperature
Sustraterock
CCA 2 (17.5%)
CCA 1 (20.1%)
Subsystem
OI
SM
Taxa
Porifera
Cnidaria
Annelida
Sipuncula
Mollusca
Arthropoda
Echinodermata
Bryozoa
Chordata

Fig. S2. (A) cluster analysis (UPGMA method) based on Bray-Curtis dissimilarity, (B) non-metric multidimensional scaling (nMDS) ordination plot based on trawl data (abundance) of the benthic megafauna, and (C) canonical correspondence (CCA). Analysis based on the community structure of benthic megafauna, grouped at major taxa level, from stations sampled at Desventuradas Islands (blue markers) and seamounts (red markers) of the Nazca Desventuradas Marine Park. Vectors in (B) and (C) represent contribution of environmental descriptors (salinity, oxygen, temperature, substrate type, depth, latitude and longitude), and ellipses in (B) represent the 95% confidence interval. Image generated using R software (version 4.0.3)[47].

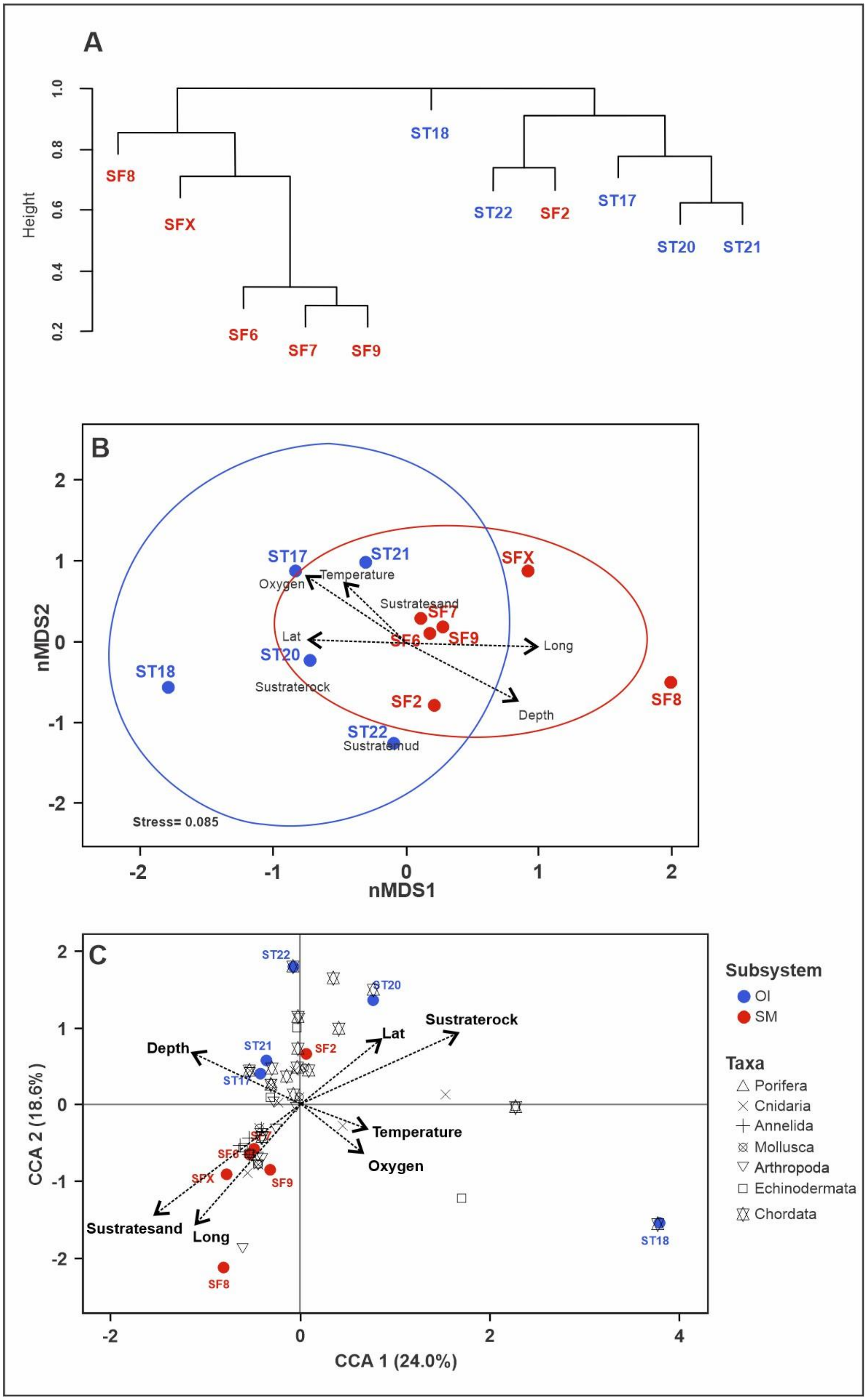

A
Height
1.0
0.8
0.6
0.4
0.2
SF8
SFX
SF6
SF7
SF9
ST18
ST22
SF2
ST17
ST20
ST21
B
ST17
ST21
SFX
Temperature
Oxygen
Sustratesand
SF7
Lat
SF6
SF9
Long
ST20
ST18
Sustraterock
SF2
SF8
Depth
ST22
Sustratemud
Stress= 0.085
nMDS2
nMDS1
C
ST22
ST20
Sustraterock
Lat
Depth
SF2
ST21
ST17
Temperature
Oxygen
SF6
SFX
SF9
Sustratesand
Long
SF8
ST18
CCA 1 (24.0%)
CCA 2 (18.6%)
Subsystem
OI
SM
Taxa
Porifera
Cnidaria
Annelida
Mollusca
Arthropoda
Echinodermata
Chordata

Fig. S3. (A) cluster analysis (UPGMA method) based on Bray-Curtis dissimilarity, (B) non-metric multidimensional scaling (nMDS) ordination plot based on ROV data (presence/absence) of the benthic megafauna, and (C) canonical correspondence (CCA). Analysis based on the community structure of benthic megafauna, grouped at major taxa level, from stations sampled at Desventuradas Islands (blue markers) and seamounts (red markers) of the Nazca Desventuradas Marine Park. Vectors in (B) and (C) represent contribution of environmental descriptors (salinity, oxygen, temperature, substrate type, depth, latitude and longitude), and ellipses in (B) represent the 95% confidence interval. Image generated using R software (version 4.0.3)[47].
