# Supplementary material for: First description of deep benthic habitats and communities of oceanic islands and seamounts of the Nazca Desventuradas Marine Park, Chile
Source: Sci Rep. 2021 Mar 18;11:6209. doi: 10.1038/s41598-021-85516-8 (PMC7973752; doi:10.1038/s41598-021-85516-8)
Supplement: Supplementary file 2 — Supplementary Legends. [file 41598_2021_85516_MOESM2_ESM.pdf]

Supplementary information

First description of deep benthic habitats and communities of oceanic islands and seamounts of the Nazca Desventuradas Marine Park, Chile.

Jan M. Tapia-Guerra, Ariadna Mecho, Erin E. Easton, María de los Ángeles Gallardo, Matthias Gorny, Javier Sellanes

Table S1 Date, depth and positions of Agassiz trawl and ROV video transects conducted on the summits of seamounts and off Desventuradas islands

Table S2 Checklist of benthic megafauna of oceanic islands and seamounts of Nazca-Desventuradas Marine Park

Table S3 Summary table of PERMANOVA analysis

Table S4 Number of habitat-forming species and number of topographic elements per site.

Figure S1 Species accumulation curves for the stations sampled within the Nazca Desventuradas Marine Park

Figure S2 Cluster analysis (UPGMA method), non-metric multidimensional scaling (nMDS) and Canonical correspondence analyses (CCA) analysis based on the community composition of benthic megafauna based in only trawl data

Figure S3 Cluster analysis (UPGMA method), non-metric multidimensional scaling (nMDS) and Canonical correspondence analyses (CCA) analysis based on the community composition of benthic megafauna based in only ROV data
